# Reliability-based design optimization for a vertical-type breakwater with multiple limit-state equations under Korean marine environments varying from sea to sea

**DOI:** 10.1038/s41598-024-59396-7

**Published:** 2024-04-20

**Authors:** Yong Jun Cho

**Affiliations:** Department of Civil Engineering, University of Seoul, Seoul, Korea

**Keywords:** Vertical type breakwater, Three-parameter Weibull distribution, Reliability-based design, Varying Korean marine environment across the sea, Nonlinear wave and lift force, Maximum likelihood estimates, Ocean sciences, Engineering

## Abstract

In this study, as part of basic research aimed at enhancing the accuracy of load and resistance coefficients to ensure their suitability for practical design and promoting the application of underutilized reliability-based design in Korea, the author conducts optimal design based on the reliability analysis of a vertical-type breakwater in the seas off of Haeundae, Yeosu, Mokpo, Gunsan, and Incheon—representative ports in Korea. In doing so, the author utilized the double-loop approach, which simultaneously addresses a reliability problem nested within an optimization process, employing the Polak–He optimization algorithm. To mitigate the substantial numerical effort required by the double-loop approach based on the Polak–He optimization algorithm, which necessitates the gradients of both cost and constraint functions, the subset simulation method was employed. In this process, the author deliberately refrained from using design waves of a specific return period and linear probabilistic models such as the Gaussian distribution, especially concerning wave and lift forces, often viewed as barriers to the widespread application of reliability-based design in Korea. Instead, the author focused on characterizing the uncertainties associated with the wave force, lift force, and overturning moment—variables that significantly impact the integrity of vertical-type breakwaters—by developing probabilistic models for these random variables directly from long-term in situ wave data. These models capture the varied characteristics of the Korean marine environment from sea to sea. In this way, the need for additional assumptions concerning the interrelationship between significant wave and maximum wave heights, along with the wave period, can be eliminated. Following Occam's razor principle, which suggests that explanations constructed with the smallest possible set of assumptions are superior, the reliability-based design optimization of a vertical-type breakwater presented in this study demonstrates promise in terms of simplicity and practicality. The limit state of the vertical-type breakwater was defined to encompass sliding, overturning, and collapse failures, and the strong interrelations between the wave force, lift force, and overturning moment were described using the Nataf joint distribution. As anticipated, simulation results show that solely considering sliding failure, as in the current reliability-based design platform in Korea, leads to an underestimated failure probability. Furthermore, ensuring a consistent failure probability for vertical-type breakwaters using design waves with a specific return period, as in past studies, is not feasible. In contrast, this study demonstrated that breakwaters optimally designed to meet the reliability index requirement of β = 3.5–4 consistently maintain a target failure probability in all sea areas.

## Introduction

**Figure 1:**
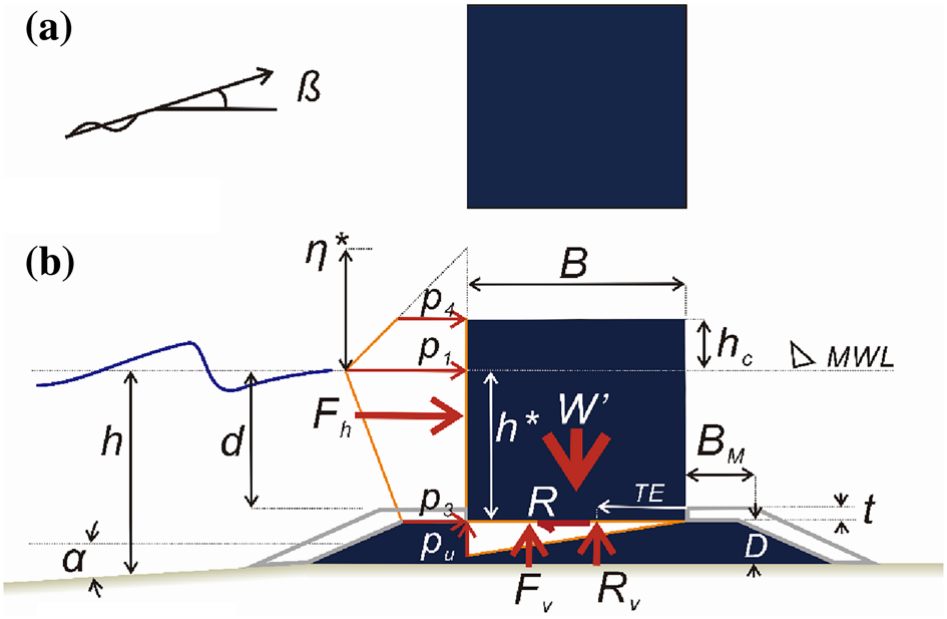
Definition sketch of the physical parameters affecting the impulsive wave force on a vertical-type breakwater using Caisson. (**a**) Plan view, (**b**) Side view

At present, vertical-type breakwaters using caissons, which are the most preferred structural type for outer port facilities in Korea, are designed to withstand waves with return periods of either 50 or 100 years. Issues related to the design waves or any errors that can arise during the subsequent design process are usually addressed by applying safety factors. In the majority of reliability-based design studies conducted in Korea, this approach has been maintained, albeit with slight modifications: the safety factor is divided into load and resistance coefficients. Despite the numerous potential applications for reliability-based design, its application in Korea remains somewhat limited^1–4^. The limited application of reliability-based design in Korea can be attributed to a lack of comprehensive efforts to integrate the diverse characteristics of the Korean marine environment into the design process^3,5,6^. This challenge is further compounded by persistent ambiguity surrounding the determination of a righteous return period. Despite the crucial role of design waves with a specific return period in the current reliability-based design platform in Korea, uncertainties persist within the Korean coastal engineering community. Design waves with a specific return period primarily focus on defining the characteristics of waves, one of the environmental factors affecting the safety of vertical-type breakwaters. These design waves do not provide any insights into the level of resilience integrated into these facilities intended to withstand such waves. The robustness of vertical-type breakwaters can be assessed through their failure probabilities, which take into account the structural response resulting from the interaction between these facilities and incoming waves^7,8^. This failure probability is pivotal in the context of reliability-based design as well. To ensure that vertical-type breakwaters designed using reliability analysis effectively fulfill their intended aim and provide adequate safety, it is crucial that the probabilistic models used in reliability analysis accurately account for the inherent irregularities in random variables. These variables, such as wave force, lift force, tidal level, friction coefficient, and overturning moment, significantly impact the structural integrity of vertical-type breakwaters (refer to Fig. 1)^9–13^. While of great importance, the current reliability-based design platform in Korea frequently relies on linear probabilistic models, such as the Gaussian distribution, particularly when dealing with wave and lift forces. Even more concerning is the treatment of these two forces as mutually independent random processes, which contradicts our physical intuition. In the current reliability-based design platform in Korea, wave and lift forces are modeled as the forces exerted by a design wave with return periods of 50 or 100 years adjusted by bias coefficients^1–4^. These bias coefficients, introduced to address the overshooting problem of Goda pressure formula, are assumed to follow their own Gaussian distribution^14^. Consequently, this common design practice implies that wave and lift forces independently follow Gaussian distributions in the ensuing reliability analysis, which raises questions about the legitimacy of the resulting load and resistance coefficients^15^. This, in turn, hinders the widespread application of reliability-based design in Korea, as these perspectives have never been addressed in past studies concerning reliability-based design conducted in Korea.

**Figure 2:**
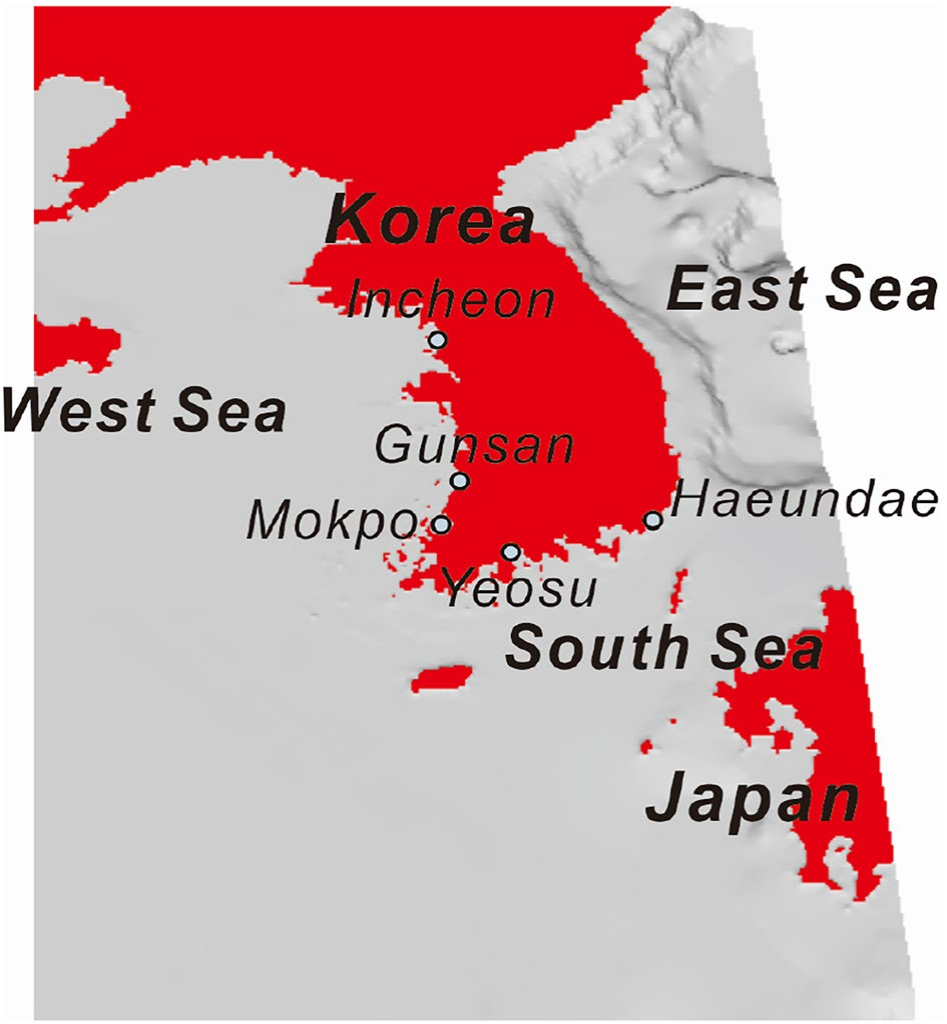
Map of the study area. The map was created using MIKE 21, a software developed by DHI Group, available at https://www.mikepoweredbydhi.com/products/mike-21-3.

To delve further into reliability-based design, each step of reliability-based design needs to be reviewed, which can be summarized as follows: Initially, define the limit state where the external and resistance forces acting on the vertical-type breakwaters are balanced. From there, the goal is to identify the combination of random variables on this limit state that has the highest probability of occurring. Once the design point is specified, we can calculate a reliability index. This index is defined as the minimum distance from the origin of the sample space to the design point, or, in other words, the failure probability. Such an approach helps distinguish between different design alternatives, ultimately providing a robust foundation for designing the target structure^16,17^. In recent years, most studies related to reliability-based design in Korea have concentrated on reverse engineering of the load and resistance coefficients of vertical-type breakwaters already in service^1,2,4^. In these studies, authors typically begin their studies by conducting reliability analyses to identify the design point. Subsequently, the load and resistance coefficients are determined, defined as the relative ratios of the values of random variables constituting the design point obtained from this process to those originally utilized in the initial design. These coefficients serve to divide the safety factor of the deterministic design into load and resistance coefficients. This approach holds significant engineering value because it enables us to assess the extent to which the design wave with a return period of 50 or 100 years, as employed in its design, deviates from adequacy or excessiveness. However, currently, the application of the aforementioned load and resistance coefficients in the design of vertical-type breakwaters in Korea is uncommon. Several factors contribute to the limited application of reliability-based design in Korea. Foremost among these factors is the lack of probabilistic models for capturing the various characteristics of the Korean marine environment from sea to sea, as discussed earlier (refer to Fig. 2). Additionally, the deficiency in the current reliability-based design platform in Korea further hampers its application. This platform primarily addresses the failure probability of a vertical-type breakwater in terms of sliding failure, neglecting critical aspects such as overturning and the collapse of a vertical-type breakwater due to exceeding the allowable bearing capacity of its foundation rock mat. Moreover, the absence of consideration of physical properties, such as the specific weight of a vertical-type breakwater and the total amount of its filler, further impedes the application of reliability-based design. A nagging question looms over the Korean coastal engineering community: Do design waves with return periods of 50 or 100 years adequately account for the inherent irregularities in wave and lifting forces, as well as overturning moments acting on vertical breakwaters? This uncertainty hinders the application of reliability-based design as well. It is noteworthy that these concerns have consistently been raised within certain factions of the Korean coastal engineering community.

In this study, as part of basic research to enhance the accuracy of load and resistance coefficients and ensure their suitability for practical design, the author conducts an optimal design based on the reliability analysis of a vertical-type breakwater using the double-loop approach. This approach consists of solving the reliability problem for each design assessed within the optimization process, employing the Polak–He optimization algorithm^18^. To achieve this, the author creates a cost function that takes into account the weight of the vertical-type breakwater along with three limit state equations for sliding, overturning, and collapse failures. The optimization is conducted within a range that maintains the failure probability within acceptable limits. During this process, the author deliberately refrained from using design waves of a specific return period and linear probabilistic models, such as a Gaussian distribution—especially for wave and lift forces, which are not free from their intrinsic limitations. The deficiencies of these linear probabilistic models become more evident under harsh wave conditions, against which vertical-type breakwaters are required to survive. These assumptions constitute the backbone of the current reliability-based design platform in Korea; however, they are often cited as barriers to the widespread application of reliability-based design in the country. Instead, the author characterized the uncertainties associated with the wave force, lift force, and overturning moment. This was achieved by utilizing probabilistic models for these random variables developed directly from long-term in situ wave data collected hourly, employing the three-parameter Weibull distribution as the underlying probability distribution. In this way, the need for extra assumptions concerning the interrelationship between significant wave and maximum wave heights, along with the wave period, as in the study by Castillo et al.^19^, can be eliminated. Following Occam's razor principle, which suggests that explanations constructed with the smallest possible set of assumptions are superior, the reliability-based design optimization of a vertical-type breakwater presented in this study shows promise in terms of simplicity and practicality. The optimization design factors include the thickness and equivalent specific weight of the vertical-type breakwater. The use of the optimized equivalent specific weight for a vertical-type breakwater provides valuable insights into estimating the physical properties and total amount of filler used in practical design. If the chosen return period is suitable, the failure probability for the vertical-type breakwater designed based on that return period should align with the failure probability achieved through optimization, where the load coefficient is equal to one. With these considerations, it becomes possible to estimate the appropriate return period by inversely engineering the yearly maximum wave force that constitutes the design point corresponding to the thickness of the optimized vertical-type breakwater, along with the probability distribution of the yearly maximum wave force. In this context, reverse engineering of the design return period corresponding to the optimized vertical-type breakwater was also conducted. These efforts aim to determine whether design waves with return periods of 50 or 100 years can effectively accommodate the inherent irregularities in random variables such as wave force, lift force, tidal level, friction coefficient, and overturning moment that act upon a vertical-type breakwater. This study is expected to make a significant contribution to resolving longstanding issues within the Korean coastal engineering community mentioned above. Furthermore, the strong interrelations between the wave force, lift force, and overturning moment are described using the Nataf joint probability distribution. As discussed earlier, this critical aspect has not been addressed in previous studies on reliability-based design in Korea, despite its substantial impact on the safety of vertical-type breakwaters. The Nataf joint distribution is renowned for its ability to represent strong correlations among random variables^20^.

## Reliability analysis

### Sliding failure

Reliability-based design assesses the probability of vertical-type breakwaters fulfilling their intended roles, referred to as reliability. The reliability function $Z$ can be defined as the difference between the resistance $R$ against the sliding of breakwaters and the wave force $S$ acting on the breakwaters. In this case, $Z$ can be written as follows:

1 $$\begin{aligned} Z & = \mu \left[ {\left( {W - \rho gdB} \right) - F_{V} } \right] - F_{H} \\ & = R - S. \\ \end{aligned}$$

To ensure the intended functionality of a vertical-type breakwater using a caisson, the reliability function must meet the following conditions:

2 $$Z > 0.$$

In Eqs. (1) and (2), $\mu$, $F_{H}$, $F_{V}$, and $d$ represent the friction coefficient, wave and lift forces acting on the breakwater, and tide level, respectively, and must be treated as probabilistic variables due to the uncertainty inherent in the marine environment. In Eq. (1), $W = \rho g\left( {{\varvec{d}} + h_{c} } \right){\varvec{B}}$ represents the weight of the breakwater, which constitutes the cost function for optimization (refer to Fig. 1), while ${\varvec{B}}$ and $\rho$ represent the thickness and equivalent density of the vertical-type breakwater, respectively, serving as two design factors.

In addition to being able to withstand sliding, as described in Eq. (2), a vertical-type breakwater must also resist overturning and ensure that the effective load $F_{e}$ on the foundation rock mat of the breaker remains below the allowable bearing capacity to fulfill its intended function.

### Overturning failure

To ensure stability against overturning, the following conditions must be met:

3 $$M_{Breaker } > M_{{F_{V} }} + M_{{F_{H} }}$$

where $M_{Breaker }$, $M_{{F_{V} }}$, and $M_{{F_{H} }}$ can be written as^21–24^;

4 $$M_{Breaker } = \left( {W - \rho gdB} \right) \times \frac{1}{2}B$$

5 $$M_{{F_{V} }} = F_{V} \times \frac{2}{3}B$$

6 $$M_{{F_{H} }} = \frac{1}{6}\left( {2 + \alpha_{3} } \right)p_{1} d^{2} + \frac{1}{2}\left( {p_{1} + p_{4} } \right)dh_{c} + \frac{1}{6}\left( {p_{1} + 2p_{4} } \right)h_{c}^{2} .$$

In Eq. (6), $p_{1}$, $p_{4}$, $h_{c}^{*}$, and $\eta^{*}$ can be written as:

7 $$p_{1} = \frac{1}{2}\left( {1 + \cos \beta } \right)\left( {\lambda_{1} \alpha_{1} + \lambda_{2} \alpha_{2} \cos^{2} \beta } \right)\gamma_{w} H_{MAX}$$

8 $$p_{4} = p_{1} \left( {1 - \frac{{h_{c} }}{\eta *}} \right),\quad \eta^{*} > h_{c} \quad$$

9 $$h_{c}^{*} = \min (\eta^{*} ,\;h_{c} )\quad$$

10 $$\eta^{*} = 0.75(1 + \cos \beta )H_{MAX} .$$

In Eqs. (6) and (7), $H_{MAX}$ is the maximum wave height, $\gamma_{w}$ is the specific weight of the water, and $\alpha_{1}$, $\alpha_{2}$, and $\alpha_{3}$ are coefficients that account for the effect of the wave period which can be written as follows:

11 $$\alpha_{1} = 0.6 + \frac{1}{2}\left( {\frac{2kh}{{\sinh 2kh}}} \right)^{2}$$

12 $$\alpha_{2} = \min \left[ {\frac{{h_{b} - d}}{{3h_{b} }}\left( {\frac{{H_{MAX} }}{d}} \right)^{2} ,\;\frac{2d}{{H_{MAX} }}} \right]$$

13 $$\alpha_{3} = 1 - \frac{{h_{c}^{*} }}{h}\left( {1 - \frac{1}{\cosh kh}} \right)$$

where $h_{b}$ denotes the water depth at site $5H_{1/3}$ away from the breakwater.

### Collapse failure

To ensure the stability of the vertical-type breakwater against collapse failure, it is imperative to maintain the effective load on the foundation rock mat below its allowable bearing capacity, as described below:

14 $$F_{e} < 490\;[{\text{N}}/{\text{m}}^{2} ]$$

where $F_{e}$, $t_{e}$, $M_{net}$, and $W_{net}$ are defined as follows:

15 $$F_{e} = \left[ {\begin{array}{*{20}l} {\frac{2}{3}\frac{{W_{net} }}{{t_{e} }}:} \hfill & {t_{e} \le \frac{B}{3}} \hfill \\ {\frac{{4W_{net} }}{B} - \frac{{6M_{net} }}{{B^{2} }}:} \hfill & {t_{e} > \frac{B}{3}} \hfill \\ \end{array} } \right.$$

16 $$t_{e} = \frac{{M_{net} }}{{W_{net} }}$$

17 $$M_{net} = M_{Breaker } - M_{{F_{V} }} - M_{{F_{H} }}$$

18 $$W_{net} = W - \rho gdB - F_{V} .$$

### Efficient Monte Carlo simulation using the subset simulation technique

The shape of the failure domain, as defined by the limit states given in Equations (2), (3), and (14), within a sample space composed of five random variables can be highly complex. In such cases, traditional analytical methods such as the First Order Reliability Method (FORM) or Second Order Reliability Method (SORM), which are preferred in Korea for reliability analysis, may not be applicable. Monte Carlo simulation can be employed for reliability analysis in these cases. However, achieving the necessary level of accuracy often requires a significant number of samples. This, in turn, results in a substantial amount of computation time, which can pose challenges when integrating it into the design process. Fortunately, recent developments, such as the subset simulation technique^25^, have emerged, which significantly reduces the number of samples by leveraging conditional probability while maintaining the necessary level of accuracy. This advancement makes Monte Carlo simulation a more efficient option for inclusion in the design process.

Utilizing conditional probability and assuming the serial occurrence of probability events (subsets, $F_{i} = \left[ {g(x) < y_{i} } \right]$) with a relatively high probability of occurrence, Au and Beck (2001) pioneered the subset simulation technique by describing the failure event $F_{i} = \left[ {g(x) < 0} \right]$ as follows:

19 $$\begin{aligned} p_{f} & = P\left[ F \right] \\ & = P\left[ {\left. {F_{m} } \right|F_{m - 1} } \right]P\left[ {F_{m - 1} } \right] \\ & \vdots \\ & = P\left[ {F_{1} } \right]\prod\limits_{i = 2}^{m} {P\left[ {\left. {F_{i} } \right|F_{i - 1} } \right]} . \\ \end{aligned}$$

In Equation (19), the threshold of each subset is selected such that it satisfies the following conditions:

20 $$F_{1} \supset F_{2} \cdots \supset F_{m} = F.$$

The occurrence probability of each subset is adjusted to range from 0.1 to 0.2. The initial threshold value is determined through a preliminary Monte Carlo simulation. For subsequent sampling at intermediate stages, the Markov chain Monte Carlo (MCMC) method, based on the modified Metropolis‒Hastings algorithm, is employed.

## Reliability-based design optimization

Reliability-based design optimization was performed to ascertain the physical properties of the breakwater, utilizing the Polak–He optimization algorithm^18^. This algorithm leverages conjugate gradients of both cost and constraint functions and is widely recognized for its capability to effectively handle local optima. The cost function comprises the weight $W = \rho_{C} g\left( {d + h_{c} } \right){\varvec{B}}$ of the vertical-type breakwater, with target reliability indices of 2, 3, 4, and 5. The optimization design factors were the breakwater thickness and the equivalent specific weight of the filler, which, when optimized, allow for the estimation of physical properties and the required amount of filler for the vertical-type breakwater. The application sites were chosen to be the seas off of Haeundae, Yeosu, Mokpo, Gunsan, and Incheon, which host Korea’s representative ports. The water depth was set at 20 m, considering the typical water depth for deployment of vertical-type breakwaters. The limit state equations $G_{i} \left[ {i = 1,2,3} \right]$ for a vertical-type breakwater consist of three equations that account for sliding, overturning, and collapse failures and can be written as follows:

21 $$\left\{ {\begin{array}{*{20}l} {G_{1} = R - S} \hfill \\\ {G_{2} = M_{Breaker } - M_{{F_{V} }} - M_{{F_{H} }} } \hfill \\\ {G_{3} = 490 - F_{e} } \hfill \\ \end{array} } \right..$$

The strong interrelations between the wave force, lift force, and overturning moment were described using the Nataf joint distribution, which is defined by marginal distributions for the wave force, lift force, and overturning moment, and the Gaussian correlation coefficient $\rho_{ij}$ between these random variables $x_{i}$^26^. The breakwater reliability problem expressed in the original space of random variables $x_{i}$ is transformed to a standard normal space $u$, where $U_{i}$ becomes an independent standard normal vector. For a Nataf joint distribution, physical random variables $x_{i}$ are transformed to correlated standard normal variables $Z_{i}$, whose correlation coefficient $\rho_{{o_{ij} }}$ obeys the following integral equation:

22 $$\rho_{ij} = \int_{ - \infty }^{\infty } {\int_{ - \infty }^{\infty } {\left( {\frac{{x_{i} - \mu_{i} }}{{\sigma_{i} }}} \right)} } \left( {\frac{{x_{j} - \mu_{j} }}{{\sigma_{j} }}} \right)\varphi \left( {z_{i} ,z_{j} ,\rho_{{o_{ij} }} } \right)dz_{i} dz_{j}$$

where $\mu_{i}$ and $\sigma_{i}$ denote the mean and standard deviation of physical random variables $x_{i}$, and $\varphi \left( {z_{i} ,z_{j} ,\rho_{{o_{ij} }} } \right)$ is the standard normal probability density function with correlation coefficient $\rho_{{o_{ij} }}$.

Independent standard normal variables $U_{i}$ are then obtained from $Z_{i}$ variables such as follows:

23 $$u = L_{o}^{ - 1} z$$

where $L_{o}^{ - 1}$ is the lower-triangular Cholesky decomposition of $R_{o} = \left[ {\rho_{{o_{ij} }} } \right]$ matrix.

The aforementioned optimization problem can be formulated as follows:

24 $$\mathop {Minimize\;W = \rho_{C} g\left( {d + h_{c} } \right){\varvec{B}}}\limits_{{\left( {\rho_{C} ,\;B} \right)}} .$$

The constraint conditions used in the numerical simulation are listed as follows:

25 $$\left\{ {\begin{array}{*{20}l} {\beta_{t} = 2,\;3,\;4,\;5} \hfill \\\ {1400 < \rho_{c} < 2100\;[{\text{kg}}/{\text{m}}^{3} ]} \hfill \\\ {10 < B < 40\;[{\text{m}}]} \hfill \\ \end{array} } \right..$$

### Probabilistic models

#### Haeundae

The probabilistic models for wave force, lift force, and overturning moment required for reliability-based design optimization were directly derived from yearly maximum time series data. These data were extracted from long-term in-situ wave data collected hourly, encompassing the varying characteristics of the Korean marine environment (refer to Fig. 2)^27^. Initially, the author compiled a database of wave and lift forces from hourly in-situ wave data spanning from January 1, 1979, to December 31, 2019, as provided by WINK^25^. In this process, the Goda pressure formula was employed to evaluate the influence of wave height and its associated period on the spatial distribution of wave force, lift force, and overturning moment^21–24^. Subsequently, a frequency analysis of 41 yearly maximum values identified from the database mentioned above was conducted. This analysis utilized the Maximum Likelihood Estimates (MLE) provided by Matlab as part of the Statistics and Machine Learning Toolbox. The aim was to derive probabilistic models for yearly maximum wave force, lift force, and overturning moment tailored to the unique features of the Korean marine environment. This approach eliminated the need for additional assumptions concerning the interrelationship between significant wave and maximum wave heights, along with the wave period, as seen in the study by Castillo et al. (2006). As a result, the reliability-based design optimization of a vertical-type breakwater presented in this study shows promise in terms of simplicity and practicality.

The tidal level was assumed to follow a normal distribution, and the standard deviation for tidal level recommended by the Japan Port and Harbor Association (JPHA)^28^ was used. According to JPHA^28^, the standard deviation of the tide level is determined by the relative size of the highest high-water level (H.H.W.L.) to the high-water level (H.W.L.). In sea areas where the H.H.W.L. similar to those of H.W.L., the standard deviation of the tidal level is $0.2\mu_{d}$. However, in cases where the H.H.W.L. exceeds twice the H.W.L., the standard deviation increases to $0.4\mu_{d}$. In the context of Korea, the relationship between the highest high-water level (H.H.W.L.) and the high-water level (H.W.L.) varies. Along the east coast, the ratio typically ranges from 2.0 to 2.5, while the south coast and west coast generally exhibit ratios between 1.0 and 1.5. The friction coefficient, which determines the scope of the resistance force in the limit state equation, is assumed to follow a normal distribution with a mean of $\mu_{\mu } = 0.636$ and a standard deviation of $\sigma_{\mu } = 0.0954$, as per Takayama^29^ and Burcharth et al.^14^, for Haeundae, Yeosu, Mokpo, Gunsan, and Incheon.

**Figure 3:**
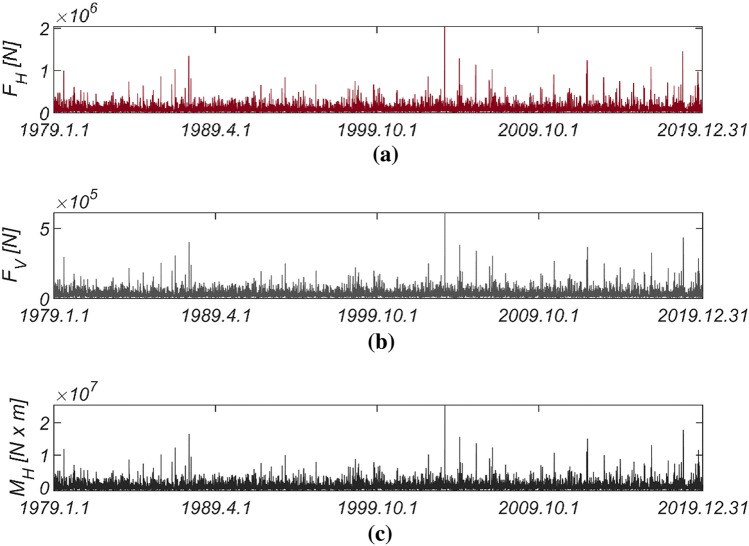
Time series of (**a**) $F_{H}$, (**b**) $F_{V}$, and (**c**) $M_{H}$ from 1979.1.1 to 2019.12.31.

**Figure 4:**
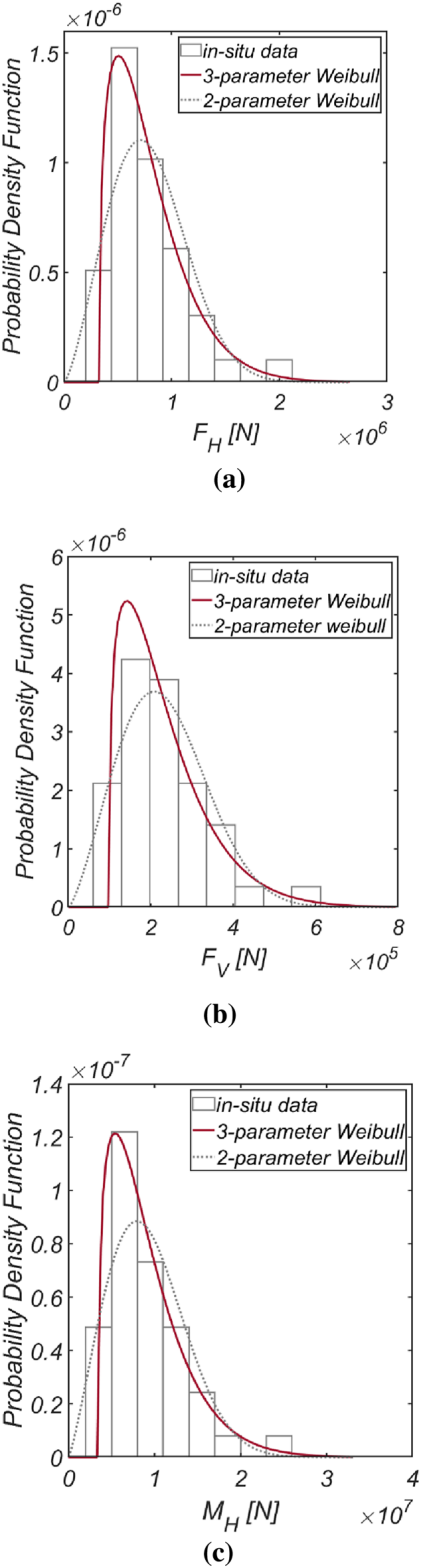
Probability density functions of the (**a**) yearly maximum wave force *F*_*H*_, (**b**) lift force *F*_*V*_, and (**c**) wave-induced moment with respect to the outer edge of the breaker *M*_*H*_[HaeunDae].

Figure 3 displays the time series data of wave and lift forces, along with the overturning moment, generated from long-term in situ wave data collected hourly in the sea off of Haeundae (refer to Fig. 2). Figure 4 illustrates the probability distributions of the yearly maximum wave force, lift force, and overturning moment. There is a significant improvement in the agreement with the measured data when employing the three-parameter Weibull distribution as the underlying probabilistic model. It is worth noting that the two-parameter Weibull distribution underestimates the occurrence probability of extreme wave and lift forces, as well as overturning moments (refer to the right tail of the distribution). The probability coefficients used in the reliability analysis are listed in Table 1, and the correlation coefficient $\rho_{ij}$ between five random variables appearing in the limit state equations defined by $G_{i} = 0$ is as follows:

26 $$\left[ {\begin{array}{*{20}c} {\rho_{{F_{H} F_{H} }} } & {\rho_{{F_{H} F_{V} }} } & {\rho_{{F_{H} M_{H} }} } & {\rho_{{F_{H} \mu }} } & {\rho_{{F_{H} d}} } \\ {\rho_{{F_{V} F_{H} }} } & {\rho_{{F_{V} F_{V} }} } & {\rho_{{F_{V} M_{H} }} } & {\rho_{{F_{V} \mu }} } & {\rho_{{F_{V} d}} } \\ {\rho_{{M_{H} F_{H} }} } & {\rho_{{M_{H} F_{V} }} } & {\rho_{{M_{H} M_{H} }} } & {\rho_{{M_{H} \mu }} } & {\rho_{{M_{H} d}} } \\ {\rho_{{\mu F_{H} }} } & {\rho_{{\mu F_{V} }} } & {\rho_{{\mu M_{H} }} } & {\rho_{\mu \mu } } & {\rho_{\mu d} } \\ {\rho_{{dF_{H} }} } & {\rho_{{dF_{V} }} } & {\rho_{{dM_{H} }} } & {\rho_{d\mu } } & {\rho_{dd} } \\ \end{array} } \right] = \left[ {\begin{array}{*{20}c} 1 & {0.997} & {0.997} & 0 & 0 \\ {0.997} & 1 & {0.997} & 0 & 0 \\ {0.997} & {0.997} & 1 & 0 & 0 \\ 0 & 0 & 0 & 1 & 0 \\ 0 & 0 & 0 & 0 & 1 \\ \end{array} } \right].$$

**Table 1:** Three-parameter Weibull distribution probability coefficients for the yearly maximum wave force $F_{H}$, lift force $F_{V}$, and wave-induced moment with respect to the outer edge of the breakwater $M_{H}$[HaeunDae].

Random variables	Scale coefficient (N)	Shape coefficient	Location coefficient (N)
Three parameter Weibull distribution			
$F_{H}$	493,397.82	1.33192	331,729.77
$F_{V}$	141,086.22	1.26522	101,039.74
$M_{H}$	6,071,597.10	1.28014	3,569,617.25

Random variables	Mean	Standard deviation	
Normal distribution			
$d$	$\mu_{d} = 18.5\;{\text{m}}$	$\sigma_{d} = 0.2\mu_{d}$	
$\mu$	0.636	$\sigma_{\mu } = 0.0954$	

#### Yeosu

**Figure 5:**
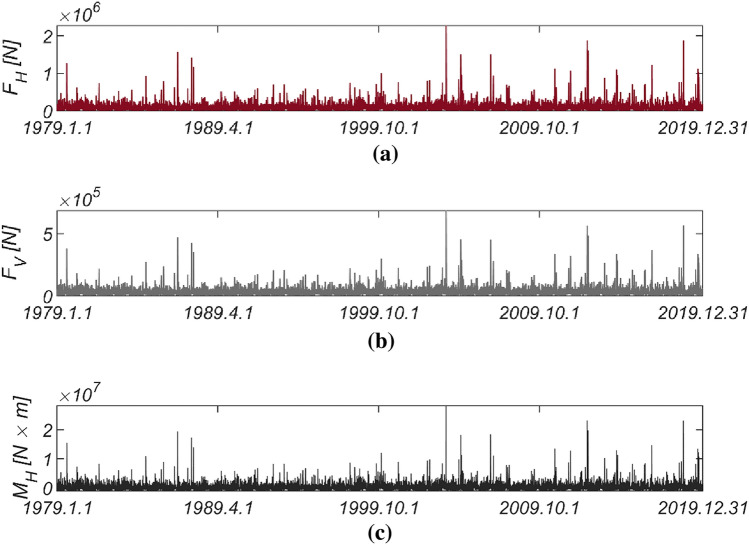
Time series of (**a**) $F_{H}$, (**b**) $F_{V}$ and (**c**) $M_{H}$ from 1979.1.1 to 2019.12.31.

**Figure 6:**
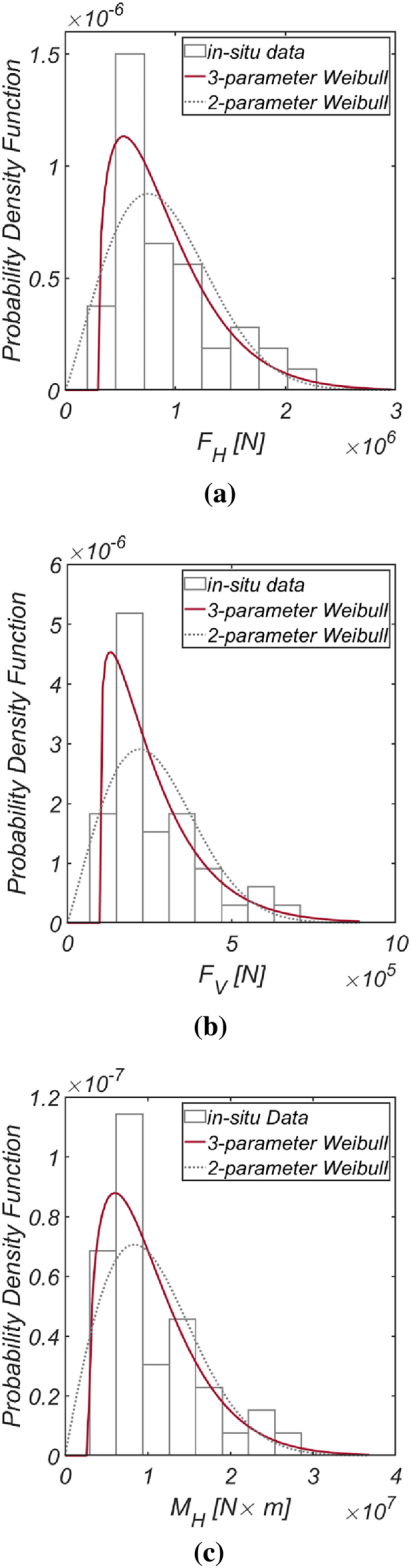
Probability density functions of the (**a**) yearly maximum wave force *F*_*H*_, (**b**) lift force *F*_*V*_, and (**c**) wave-induced moment with respect to the outer edge of the breaker *M*_*H*_ [Yeosu].

Figure 5 displays the time series data of wave and lift forces, along with the overturning moment generated from long-term in-situ wave data collected hourly in the sea off of Yeosu (refer to Fig. 2). This data was obtained using the same methodology as previously studied in Haeundae, covering the period from January 1, 1979, to December 31, 2019. In Fig. 6, the probability distributions of the yearly maximum wave force, lift force, and overturning moment extracted from 41 yearly maximum values using the same approach as in Haeundae are depicted. Similar to Haeundae, the best agreement with the measured data was achieved by employing the three-parameter Weibull distribution as the underlying probability model. It was observed, as in Haeundae, that the two-parameter Weibull distribution underestimates the occurrence probability of extreme wave and lifting forces, as well as overturning moments (refer to the right tail of the distribution). The probability coefficients used in the reliability analysis are listed in Table 2, and the correlation coefficient $\rho_{ij}$ between five random variables appearing in the limit state equations defined by $G_{i} = 0$ is as follows:

27 $$\left[ {\begin{array}{*{20}c} {\rho_{{F_{H} F_{H} }} } & {\rho_{{F_{H} F_{V} }} } & {\rho_{{F_{H} M_{H} }} } & {\rho_{{F_{H} \mu }} } & {\rho_{{F_{H} d}} } \\ {\rho_{{F_{V} F_{H} }} } & {\rho_{{F_{V} F_{V} }} } & {\rho_{{F_{V} M_{H} }} } & {\rho_{{F_{V} \mu }} } & {\rho_{{F_{V} d}} } \\ {\rho_{{M_{H} F_{H} }} } & {\rho_{{M_{H} F_{V} }} } & {\rho_{{M_{H} M_{H} }} } & {\rho_{{M_{H} \mu }} } & {\rho_{{M_{H} d}} } \\ {\rho_{{\mu F_{H} }} } & {\rho_{{\mu F_{V} }} } & {\rho_{{\mu M_{H} }} } & {\rho_{\mu \mu } } & {\rho_{\mu d} } \\ {\rho_{{dF_{H} }} } & {\rho_{{dF_{V} }} } & {\rho_{{dM_{H} }} } & {\rho_{d\mu } } & {\rho_{dd} } \\ \end{array} } \right] = \left[ {\begin{array}{*{20}c} 1 & {0.997} & {0.997} & 0 & 0 \\ {0.997} & 1 & {0.997} & 0 & 0 \\ {0.997} & {0.997} & 1 & 0 & 0 \\ 0 & 0 & 0 & 1 & 0 \\ 0 & 0 & 0 & 0 & 1 \\ \end{array} } \right].$$

**Table 2:** List of probability coefficients of the three-parameter Weibull distribution for the yearly maximum wave force $F_{H}$, lift force $F_{V}$, and wave-induced moment with respect to the outer edge of the breaker $M_{H}$ [Yeosu].

Random variables	Scale coefficient (N)	Shape coefficient	Location coefficient (N)
Three parameter Weibull distribution			
$F_{H}$	647,980.39	1.33380	301,243.04
$F_{V}$	171,188.49	1.14446	103,139.74
$M_{H}$	8,343,721.39	1.35150	2,943,208.17

Random variables	Mean	Standard deviation	
Normal distribution			
$d$	$\mu_{d} = 18.5\;{\text{m}}$	$\sigma_{d} = 0.2\mu_{d}$	
$\mu$	0.636	$\sigma_{\mu } = 0.0954$	

#### Mokpo

**Figure 7:**
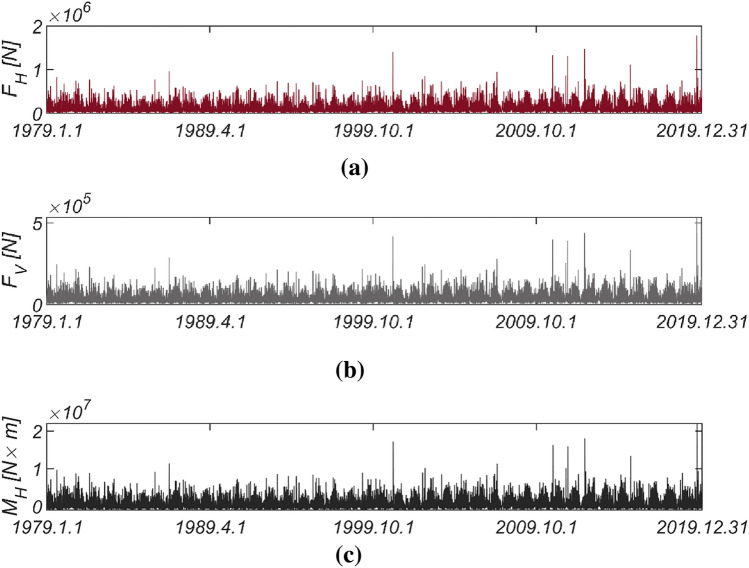
Time series of (**a**) $F_{H}$, (**b**) $F_{V}$ and (**c**) $M_{H}$ from 1979.1.1 to 2019.12.31.

**Figure 8:**
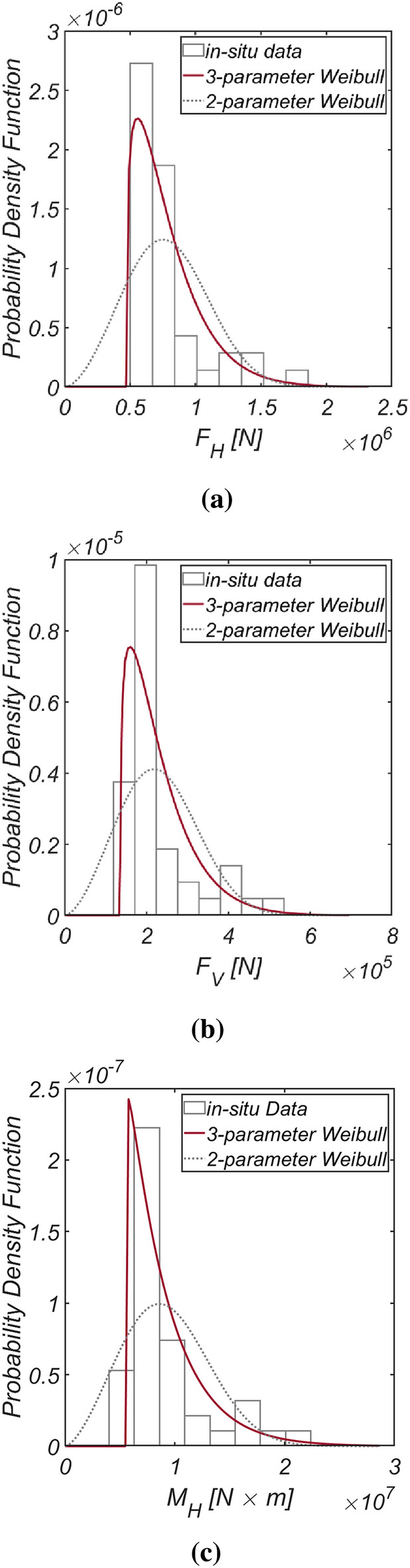
Probability density functions of the (**a**) yearly maximum wave force *F*_*H*_, (**b**) lift force *F*_*V*_, and (**c**) wave-induced moment with respect to the outer edge of the breaker *M*_*H*_ [Mokpo].

Figure 7 presents the time series data of wave and lift forces, along with the overturning moment generated from long-term in situ wave data collected hourly in the sea off of Mokpo (refer to Fig. 2), following the same approach as previously studied in Haeundae and Yeosu. This dataset spans from January 1, 1979, to December 31, 2019. Additionally, Fig. 8 shows the probability distributions of the yearly maximum wave force, lift force, and overturning moment extracted from 41 yearly maximum values using the same method as applied in Haeundae and Yeosu. Like the results obtained in Haeundae and Yeosu, good agreement with the measured data is achieved when employing the three-parameter Weibull distribution as the underlying probability model. The probability coefficients used in the reliability analysis are listed in Table 3. The probability coefficients used in the reliability analysis are listed in Table 3, and the correlation coefficient $\rho_{ij}$ between five random variables appearing in the limit state equations defined by $G_{i} = 0$ is as follows:

28 $$\left[ {\begin{array}{*{20}c} {\rho_{{F_{H} F_{H} }} } & {\rho_{{F_{H} F_{V} }} } & {\rho_{{F_{H} M_{H} }} } & {\rho_{{F_{H} \mu }} } & {\rho_{{F_{H} d}} } \\ {\rho_{{F_{V} F_{H} }} } & {\rho_{{F_{V} F_{V} }} } & {\rho_{{F_{V} M_{H} }} } & {\rho_{{F_{V} \mu }} } & {\rho_{{F_{V} d}} } \\ {\rho_{{M_{H} F_{H} }} } & {\rho_{{M_{H} F_{V} }} } & {\rho_{{M_{H} M_{H} }} } & {\rho_{{M_{H} \mu }} } & {\rho_{{M_{H} d}} } \\ {\rho_{{\mu F_{H} }} } & {\rho_{{\mu F_{V} }} } & {\rho_{{\mu M_{H} }} } & {\rho_{\mu \mu } } & {\rho_{\mu d} } \\ {\rho_{{dF_{H} }} } & {\rho_{{dF_{V} }} } & {\rho_{{dM_{H} }} } & {\rho_{d\mu } } & {\rho_{dd} } \\ \end{array} } \right] = \left[ {\begin{array}{*{20}c} 1 & {0.997} & {0.997} & 0 & 0 \\ {0.997} & 1 & {0.997} & 0 & 0 \\ {0.997} & {0.997} & 1 & 0 & 0 \\ 0 & 0 & 0 & 1 & 0 \\ 0 & 0 & 0 & 0 & 1 \\ \end{array} } \right].$$

**Table 3:** List of probability coefficients of the three-parameter Weibull distribution for the yearly maximum wave force $F_{H}$, lift force $F_{V}$, and wave-induced moment with respect to the outer edge of the breaker $M_{H}$ [Mokpo].

Random variables	Scale coefficient (N)	Shape coefficient	Location coefficient (N)
Three parameter Weibull distribution			
$F_{H}$	331,080.65	1.20989	478,381.12
$F_{V}$	99,844.53	1.19616	137,864.19
$M_{H}$	3,682,282.53	1.03357	5,602,928.97

Random Variables	Mean	Standard Deviation	
Normal distribution			
$d$	$\mu_{d} = 18.5\;{\text{m}}$	$\sigma_{d} = 0.2\mu_{d}$	
$\mu$	0.636	$\sigma_{\mu } = 0.0954$	

#### Gunsan

**Figure 9:**
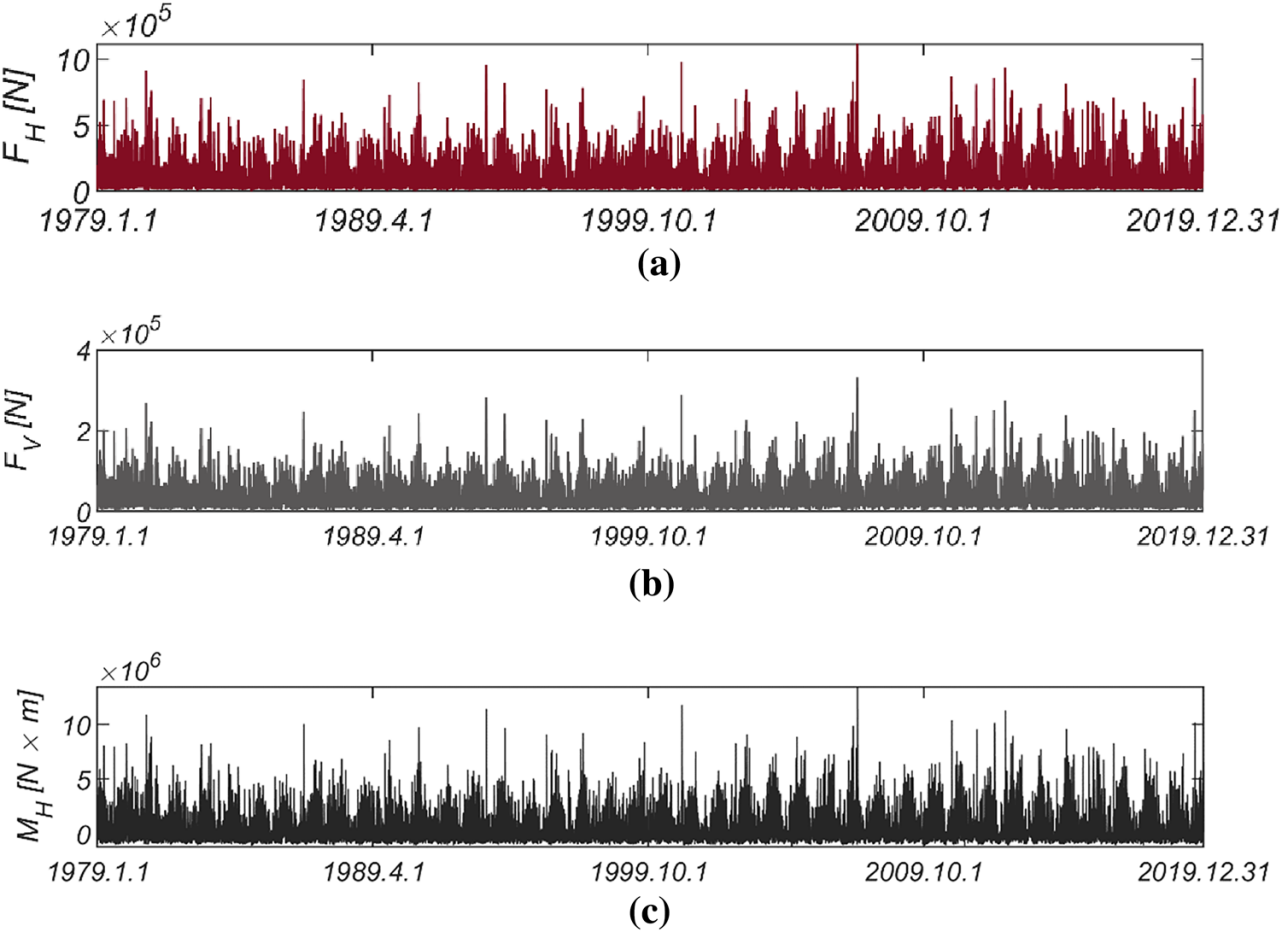
Time series of (**a**) $F_{H}$, (**b**) $F_{V}$ and (**c**) $M_{H}$ from 1979.1.1 to 2019.12.31.

**Figure 10:**
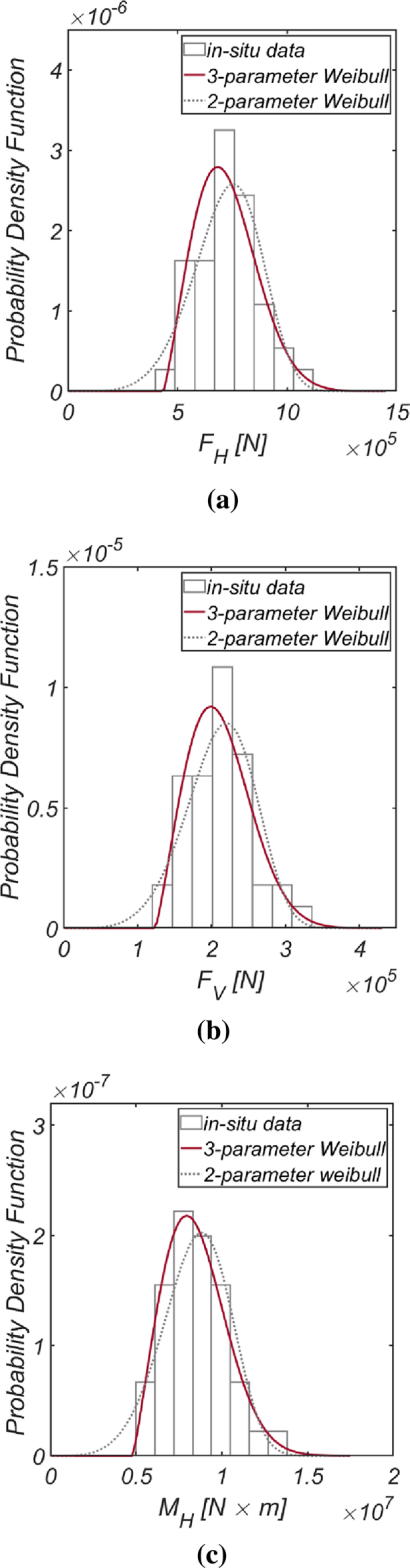
Probability density functions of the (**a**) yearly maximum wave force *F*_*H*_, (**b**) lift force *F*_*V*_, and (**c**) wave-induced moment with respect to the outer edge of the breaker *M*_*H*_ [Gunsan].

Figure 9 shows the time series data of wave and lift forces, along with the overturning moment generated from long-term in-situ wave data collected hourly in the sea off of Gunsan (refer to Fig. 2). Additionally, Fig. 10 shows the probability distributions of the yearly maximum wave force, lifting force, and overturning moment extracted from 41 yearly maximum values using the same methods applied in Haeundae, Yeosu, and Mokpo. Unlike in Haeundae, Yeosu, and Mokpo, good agreement with the measured data is attained when utilizing the two-parameter Weibull distribution as the underlying probability model. The probability coefficients used in the reliability analysis are listed in Table 4, and the correlation coefficient $\rho_{ij}$ between five random variables appearing in the limit state equations defined by $G_{i} = 0$ is as follows:

29 $$\left[ {\begin{array}{*{20}c} {\rho_{{F_{H} F_{H} }} } & {\rho_{{F_{H} F_{V} }} } & {\rho_{{F_{H} M_{H} }} } & {\rho_{{F_{H} \mu }} } & {\rho_{{F_{H} d}} } \\ {\rho_{{F_{V} F_{H} }} } & {\rho_{{F_{V} F_{V} }} } & {\rho_{{F_{V} M_{H} }} } & {\rho_{{F_{V} \mu }} } & {\rho_{{F_{V} d}} } \\ {\rho_{{M_{H} F_{H} }} } & {\rho_{{M_{H} F_{V} }} } & {\rho_{{M_{H} M_{H} }} } & {\rho_{{M_{H} \mu }} } & {\rho_{{M_{H} d}} } \\ {\rho_{{\mu F_{H} }} } & {\rho_{{\mu F_{V} }} } & {\rho_{{\mu M_{H} }} } & {\rho_{\mu \mu } } & {\rho_{\mu d} } \\ {\rho_{{dF_{H} }} } & {\rho_{{dF_{V} }} } & {\rho_{{dM_{H} }} } & {\rho_{d\mu } } & {\rho_{dd} } \\ \end{array} } \right] = \left[ {\begin{array}{*{20}c} 1 & {0.997} & {0.997} & 0 & 0 \\ {0.997} & 1 & {0.997} & 0 & 0 \\ {0.997} & {0.997} & 1 & 0 & 0 \\ 0 & 0 & 0 & 1 & 0 \\ 0 & 0 & 0 & 0 & 1 \\ \end{array} } \right].$$

**Table 4:** List of probability coefficients of the three-parameter Weibull distribution for the yearly maximum wave force $F_{H}$, lift force $F_{V}$, and wave-induced moment with respect to the outer edge of the breaker $M_{H}$ [Gunsan].

Random variables	Scale coefficient (N)	Shape coefficient	Location coefficient (N)
Three parameter Weibull distribution			
$F_{H}$	326,887.93	2.18946	436,474.79
$F_{V}$	99,184.69	2.18795	123,956.31
$M_{H}$	4,180,039.83	2.1817	4,792,302.16

Random variables	Scale coefficient (N)	Shape coefficient	
Two parameter Weibull distribution			
$F_{H}$	784,358.42	5.39834	
$F_{V}$	229,357.57	5.19711	
$M_{H}$	9,227,254.59	4.96215	

Random variables	Mean	Standard deviation	
Normal distribution			
$d$	$\mu_{d} = 18.5\;{\text{m}}$	$\sigma_{d} = 0.2\mu_{d}$	
$\mu$	0.636	$\sigma_{\mu } = 0.0954$	

#### Incheon

**Figure 11:**
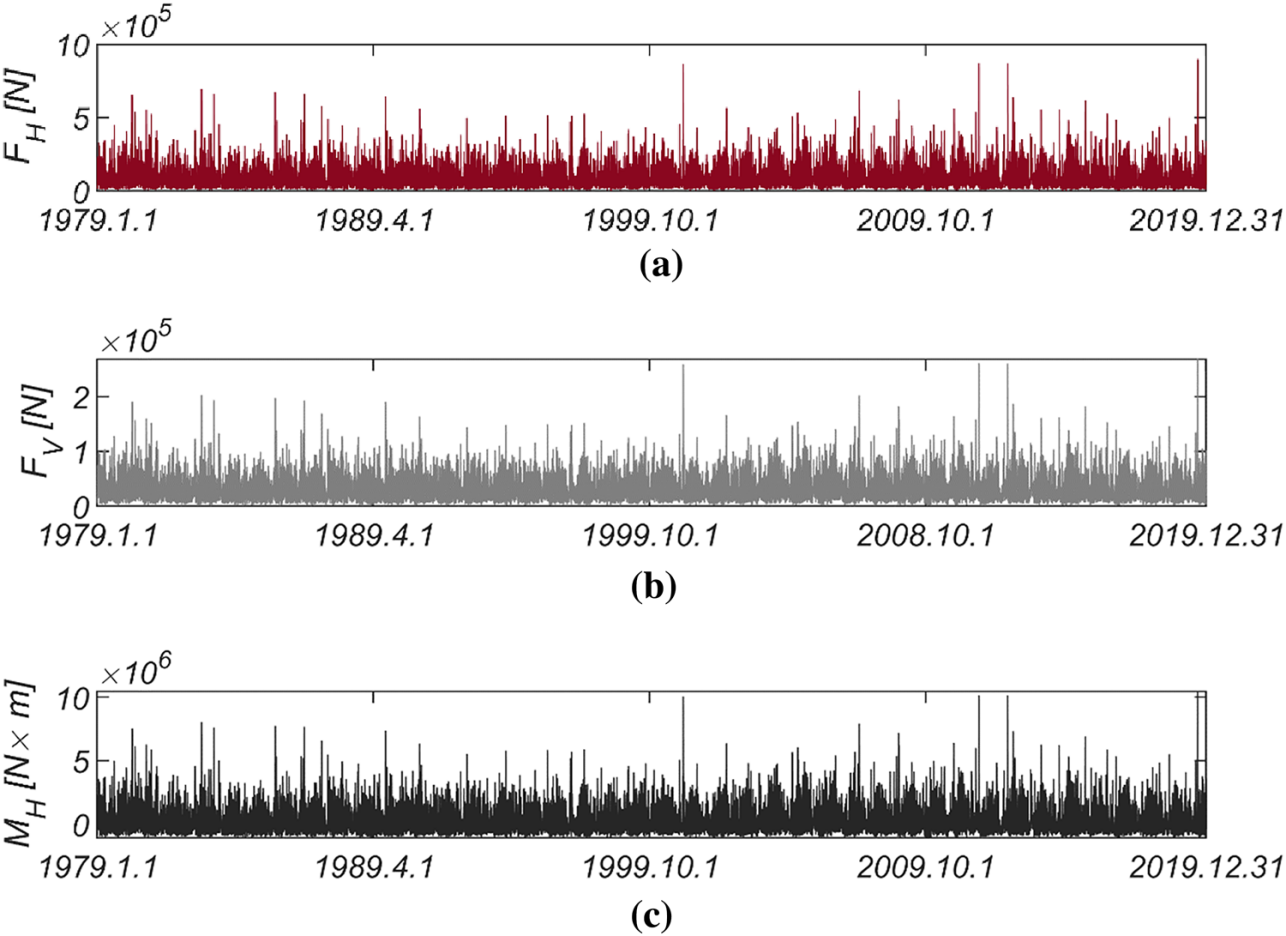
Time series of (**a**) $F_{H}$, (**b**) $F_{V}$ and (**c**) $M_{H}$ from 1979.1.1 to 2019.12.31.

**Figure 12:**
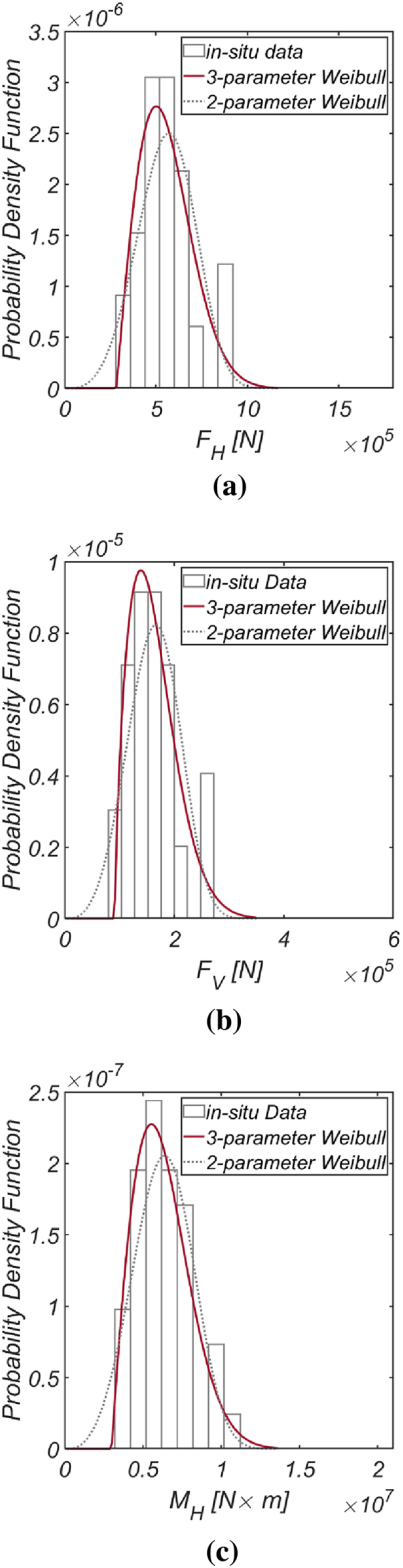
Probability density functions of the (**a**) yearly maximum wave force *F*_*H*_, (**b**) lift force *F*_*V*_, and (**c**) wave-induced moment with respect to the outer edge of the breaker *M*_*H*_ [Incheon].

In Fig. 11, the time series data of wave and lift forces, along with the overturning moment generated from long-term in-situ wave data collected hourly in the sea off of Incheon (refer to Fig. 2), is depicted. Additionally, Fig. 12 displays the probability distributions of the yearly maximum wave force, lifting force, and overturning moment extracted from 41 yearly maximum values using the same methods applied in Haeundae, Yeosu, Mokpo, and Gunsan. Unlike in Haeundae, Yeosu, and Mokpo, good agreement with the measured data is attained when utilizing the two-parameter Weibull distribution as the underlying probability model. The probability coefficients used in the reliability analysis are listed in Table 5, and the correlation coefficient $\rho_{ij}$ between five random variables appearing in the limit state equations defined by $G_{i} = 0$ is as follows:

30 $$\left[ {\begin{array}{*{20}c} {\rho_{{F_{H} F_{H} }} } & {\rho_{{F_{H} F_{V} }} } & {\rho_{{F_{H} M_{H} }} } & {\rho_{{F_{H} \mu }} } & {\rho_{{F_{H} d}} } \\ {\rho_{{F_{V} F_{H} }} } & {\rho_{{F_{V} F_{V} }} } & {\rho_{{F_{V} M_{H} }} } & {\rho_{{F_{V} \mu }} } & {\rho_{{F_{V} d}} } \\ {\rho_{{M_{H} F_{H} }} } & {\rho_{{M_{H} F_{V} }} } & {\rho_{{M_{H} M_{H} }} } & {\rho_{{M_{H} \mu }} } & {\rho_{{M_{H} d}} } \\ {\rho_{{\mu F_{H} }} } & {\rho_{{\mu F_{V} }} } & {\rho_{{\mu M_{H} }} } & {\rho_{\mu \mu } } & {\rho_{\mu d} } \\ {\rho_{{dF_{H} }} } & {\rho_{{dF_{V} }} } & {\rho_{{dM_{H} }} } & {\rho_{d\mu } } & {\rho_{dd} } \\ \end{array} } \right] = \left[ {\begin{array}{*{20}c} 1 & {0.997} & {0.997} & 0 & 0 \\ {0.997} & 1 & {0.997} & 0 & 0 \\ {0.997} & {0.997} & 1 & 0 & 0 \\ 0 & 0 & 0 & 1 & 0 \\ 0 & 0 & 0 & 0 & 1 \\ \end{array} } \right].$$

**Table 5:** List of probability coefficients of the three-parameter Weibull distribution for the yearly maximum wave force $F_{H}$, lift force $F_{V}$, and wave-induced moment with respect to the outer edge of the breaker $M_{H}$ [Incheon].

Random variables	Scale coefficient (N)	Shape coefficient	Location coefficient (N)
Three parameter Weibull distribution			
$F_{H}$	309,816.08	1.99796	282,273.38
$F_{V}$	80,112.89	1.70323	91,393.08
$M_{H}$	3,692,112.39	1.93576	3,003,843.88

Random variables	Scale coefficient (N)	Shape coefficient	
Two parameter Weibull distribution			
$F_{H}$	612,751.48	4.02957	
$F_{V}$	179,578.32	3.87331	
$M_{H}$	6,944,819.66	3.72731	

Random variables	Mean	Standard deviation	
Normal distribution			
$d$	$\mu_{d} = 18.5\;{\text{m}}$	$\sigma_{d} = 0.2\mu_{d}$	
$\mu$	0.636	$\sigma_{\mu } = 0.0954$	

### Reliability analysis

To demonstrate the impact of the newly introduced overturning and collapse failure modes in this study on the safety of vertical-type breakwaters, the author first conducted reliability analyses for Haeundae and Gunsan, representing the South and West Seas (refer to Fig. 2). These analyses entailed adjusting the thickness of a vertical-type breakwater and evaluating different combinations of failure modes before proceeding with reliability-based design optimization. In this process, any occurrence where any one of the three failure modes is observed is considered a failure.

#### Haeundae

**Figure 13:**
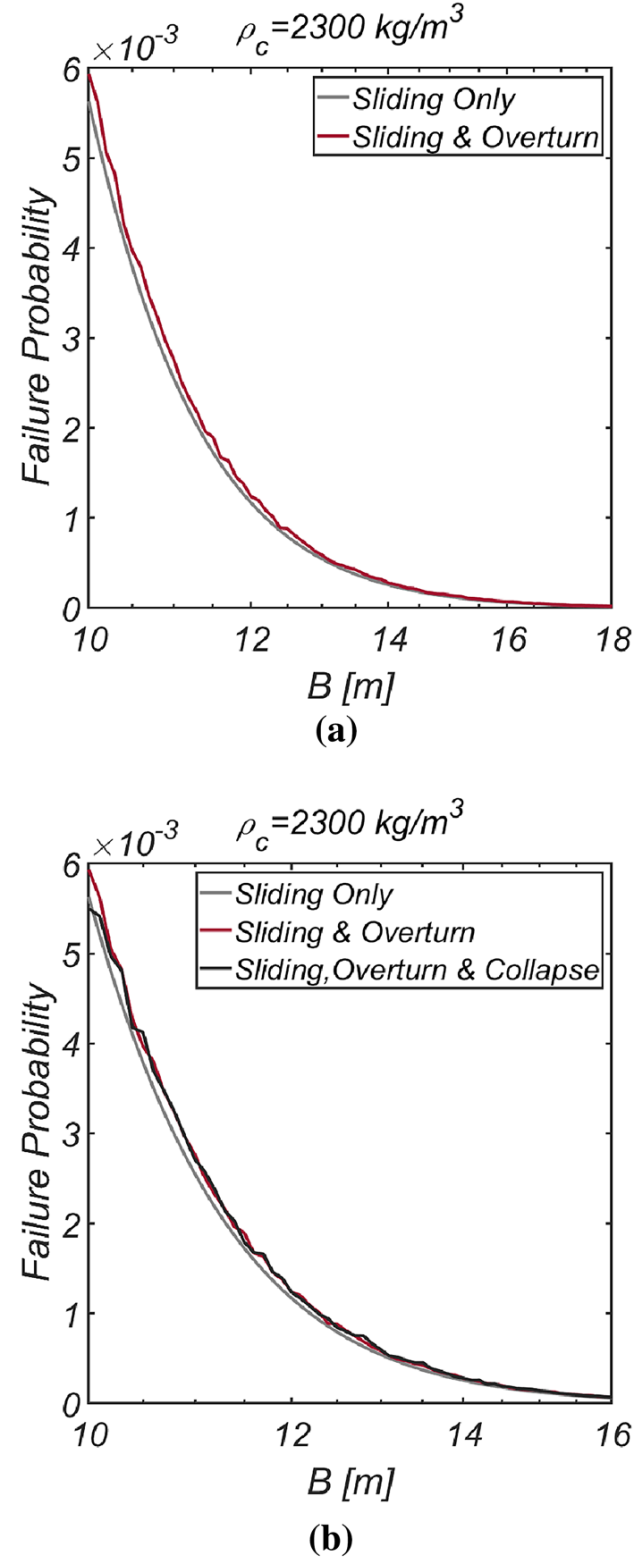
Failure probability of a vertical breaker with varying caisson thickness for $\rho_{C} = 2300\;{\text{kg}}/{\text{m}}^{3}$. (**a**) Sliding and overturn, (**b**) Sliding, overturning and collapse.

The reliability analysis results for varying breakwater thicknesses are depicted in Fig. 13. As anticipated, the failure probability decreased as the vertical-type breakwater thickness increased. Notably, relying solely on sliding failure resulted in an underestimation of the failure probability by 6.2% for the case of B=11m, despite sliding being identified as a primary failure mode of vertical-type breakwaters, as asserted by the current reliability-based design platform in Korea.

**Figure 14:**
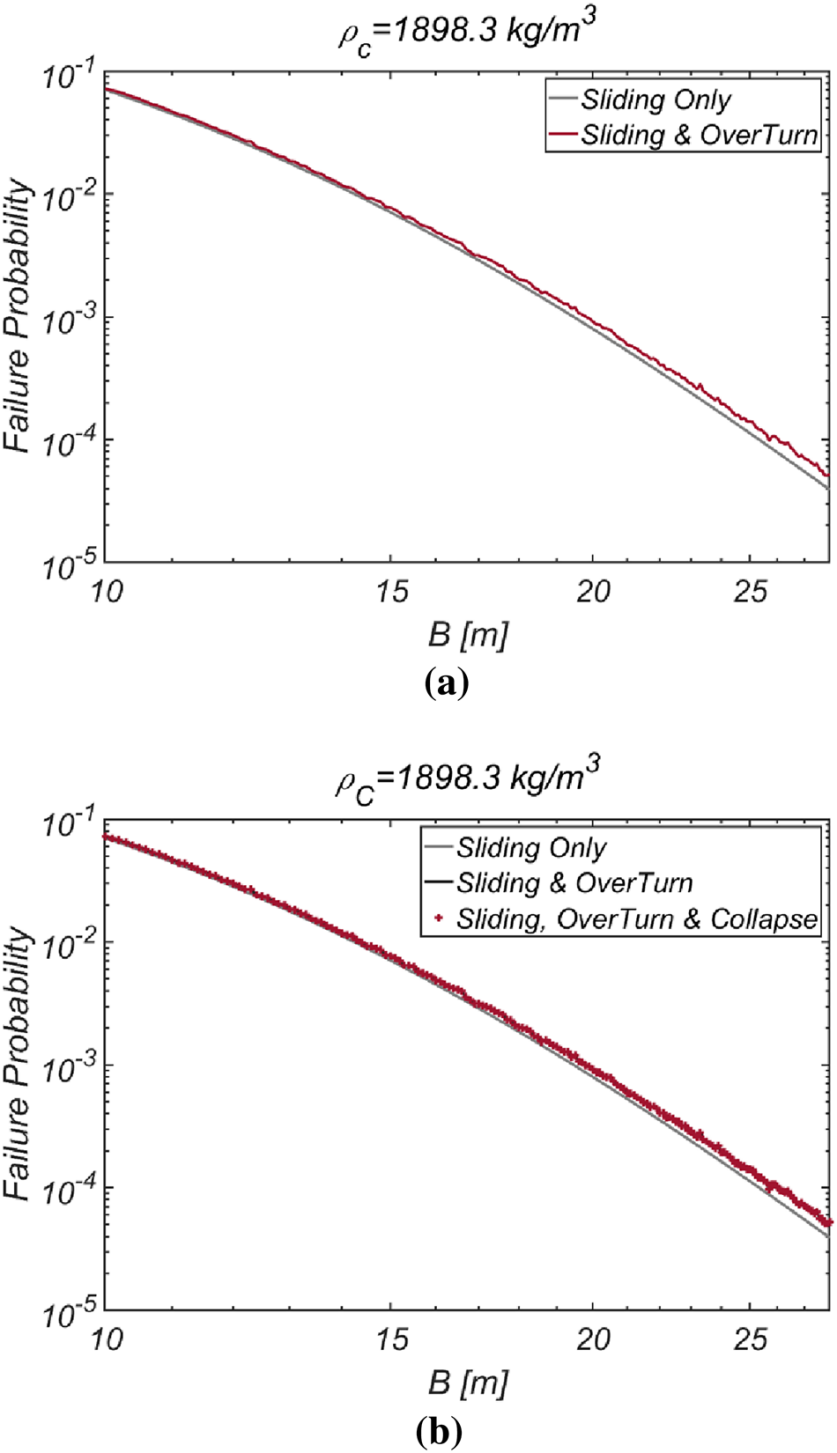
Failure probability of a vertical breaker with varying caisson thickness for $\rho_{C} = 1898.3\;{\text{kg}}/{\text{m}}^{3}$. (**a**) Sliding and overturning, (**b**) Sliding, overturning and collapse.

**Figure 15:**
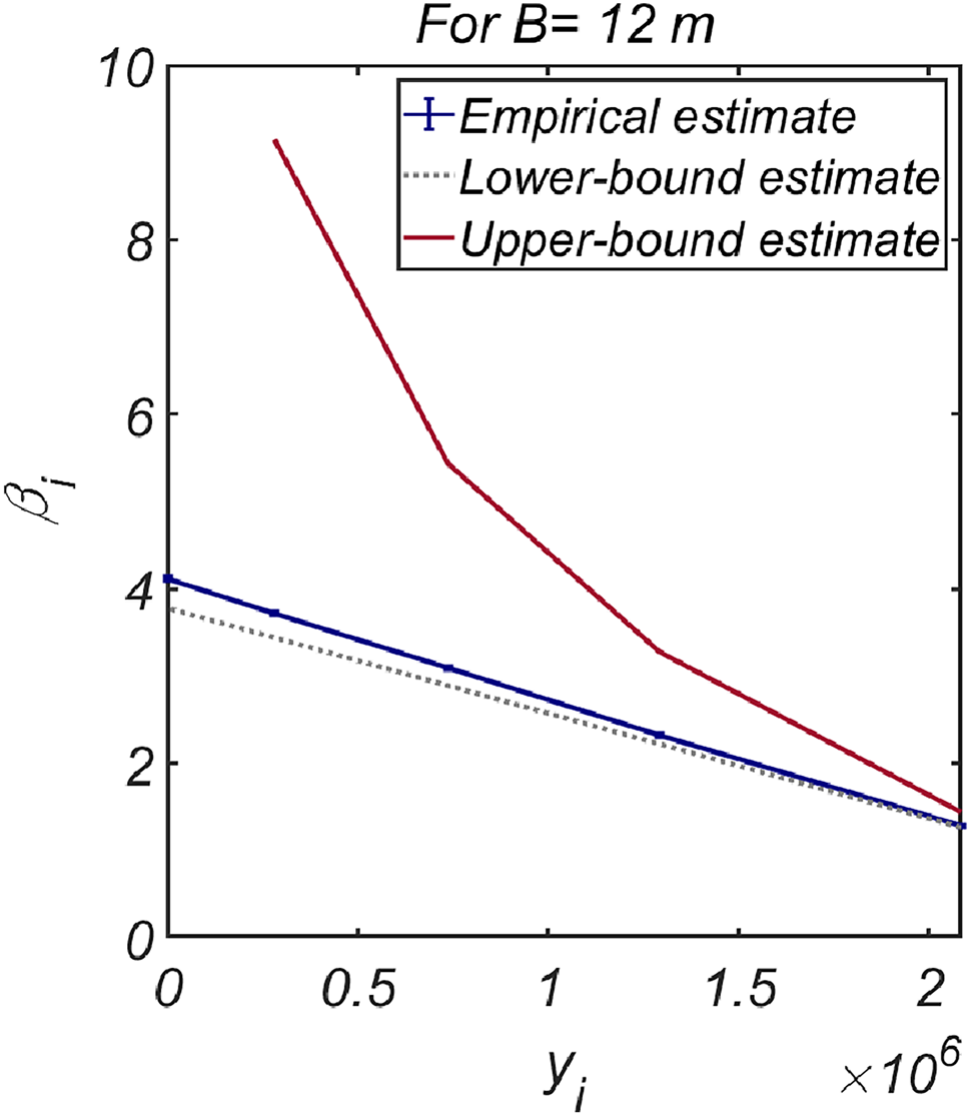
Subset simulation results.

**Figure 16:**
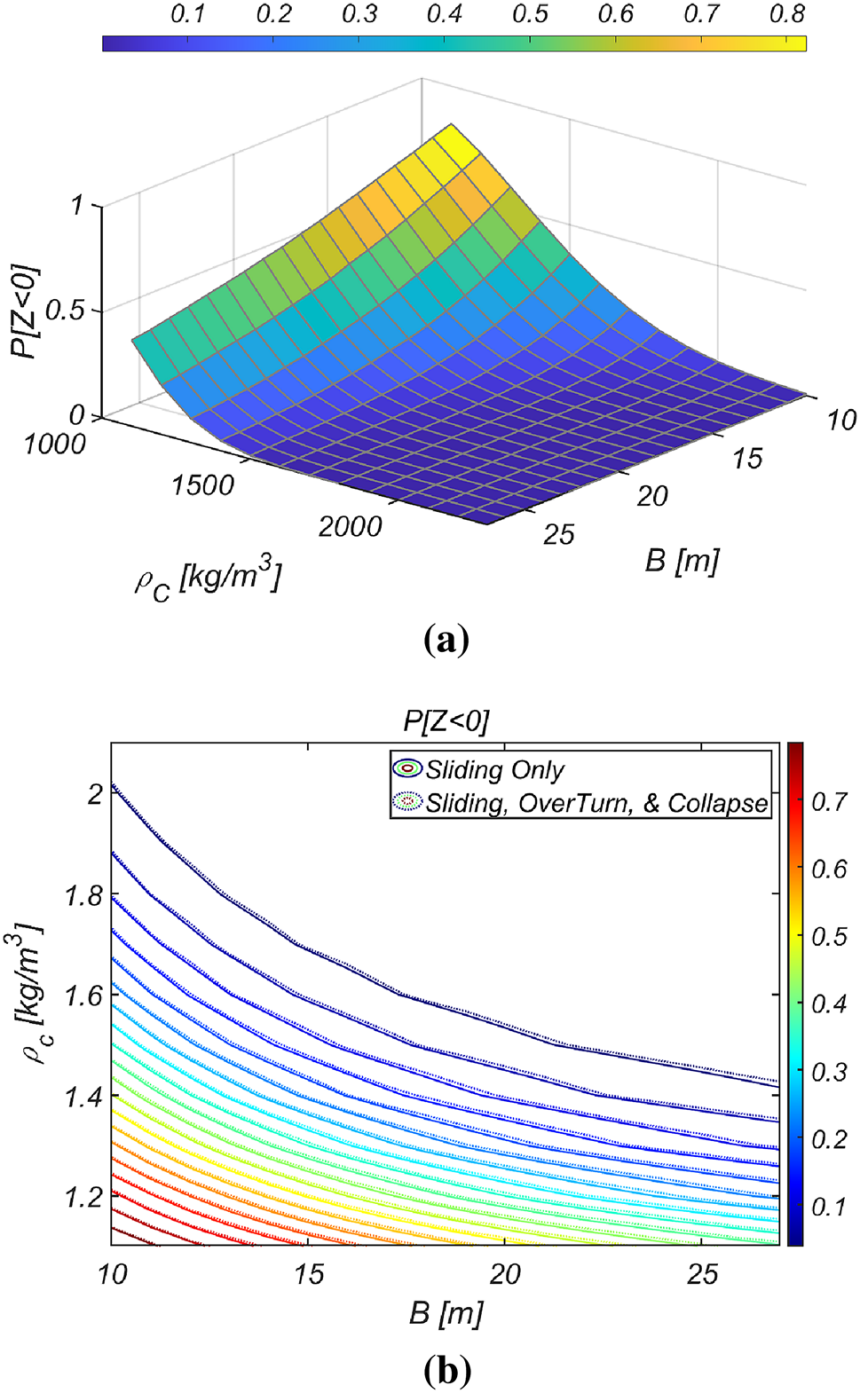
Contour plot of the failure probability of a vertical breaker for various $B$ and $\rho_{C}$. (**a**) 3D, (**b**) 2D.

Figure 14 shows the reliability analysis results for a lighter vertical-type breakwater with a density of $\rho_{c} = 1898.3\;{\text{kg}}/{\text{m}}^{3}$. It is evident that the likelihood of breakwater failure due to overturning increases with increasing breakwater thickness owing to the rising lifting force as the breakwater becomes thicker. However, the contribution of collapse failure mode was found to be insignificant in the case of the lightened breakwater. These simulation results align with our physical intuition and imply that vertical-type breakwaters might be under-designed if sliding and overturning are treated as mutually independent failure modes, as in the current reliability-based design platform. Therefore, an amendment seems inevitable. In Fig. 15, the reliability indices β are gradually corrected as the number of subsets increases in the Monte Carlo simulation using the subset simulation technique. Figure 16 shows a contour plot of the failure probability simulated by varying the thickness and equivalent density of the vertical-type breakwater. As expected, the failure probability decreases with increasing thickness and equivalent density of the vertical-type breakwater.

#### Gunsan

**Figure 17:**
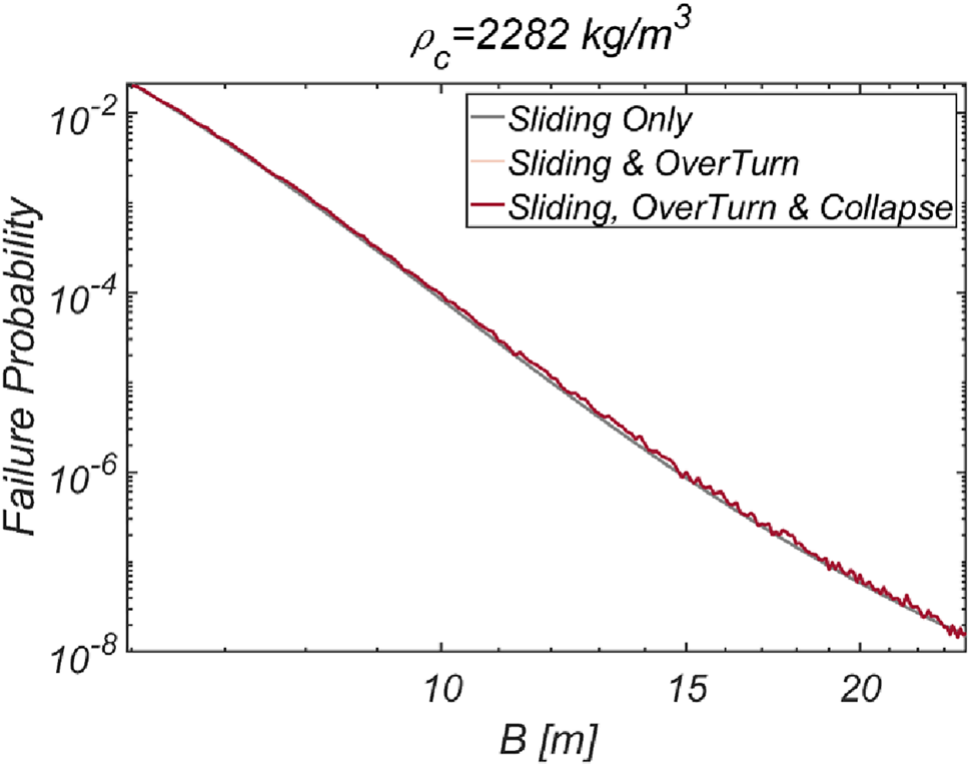
Failure probability of a vertical breaker with varying caisson thickness for $\rho_{C} = 2282\;{\text{kg}}/{\text{m}}^{3}$.

**Figure 18:**
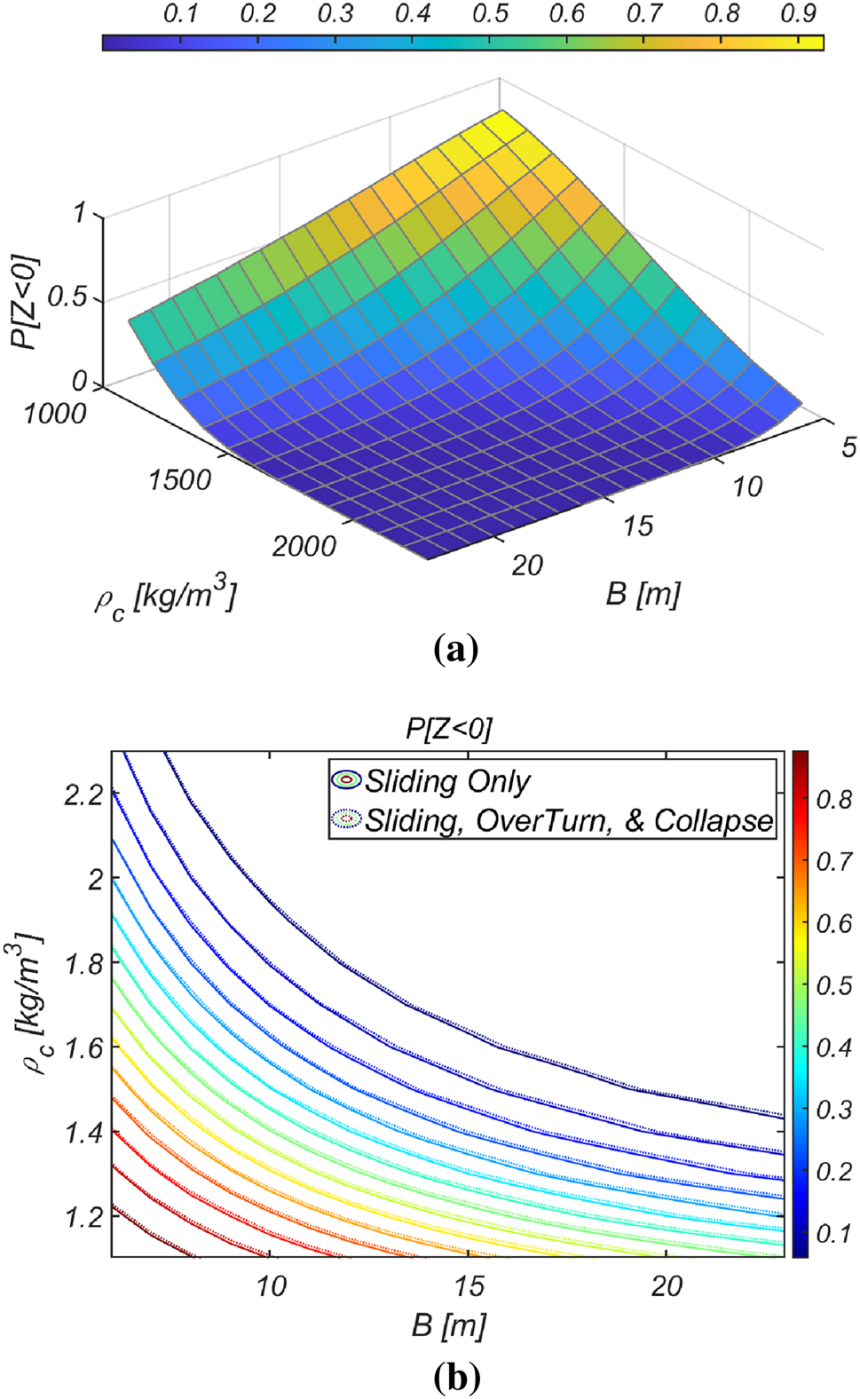
Contour plot of the failure probability of a vertical breaker for various $B$ and $\rho_{C}$. (**a**) 3D, (**b**) 2D.

The reliability analysis results obtained by varying the breakwater thickness are shown in Fig. 17. As expected, the failure probability decreased with increasing vertical-type breakwater thickness. However, due to the relatively mild marine environment in the sea off of Gunsan, the contributions of overturning and collapse failure mode to the failure probability appear insignificant. Figure 18 shows a contour plot of the failure probability simulated by varying the thickness and equivalent density of the vertical-type breakwater. As expected, the failure probability clearly decreases as the thickness and equivalent density of the vertical-type breakwater increase.

### Reliability-based design optimization

#### Haeundae

**Figure 19:**
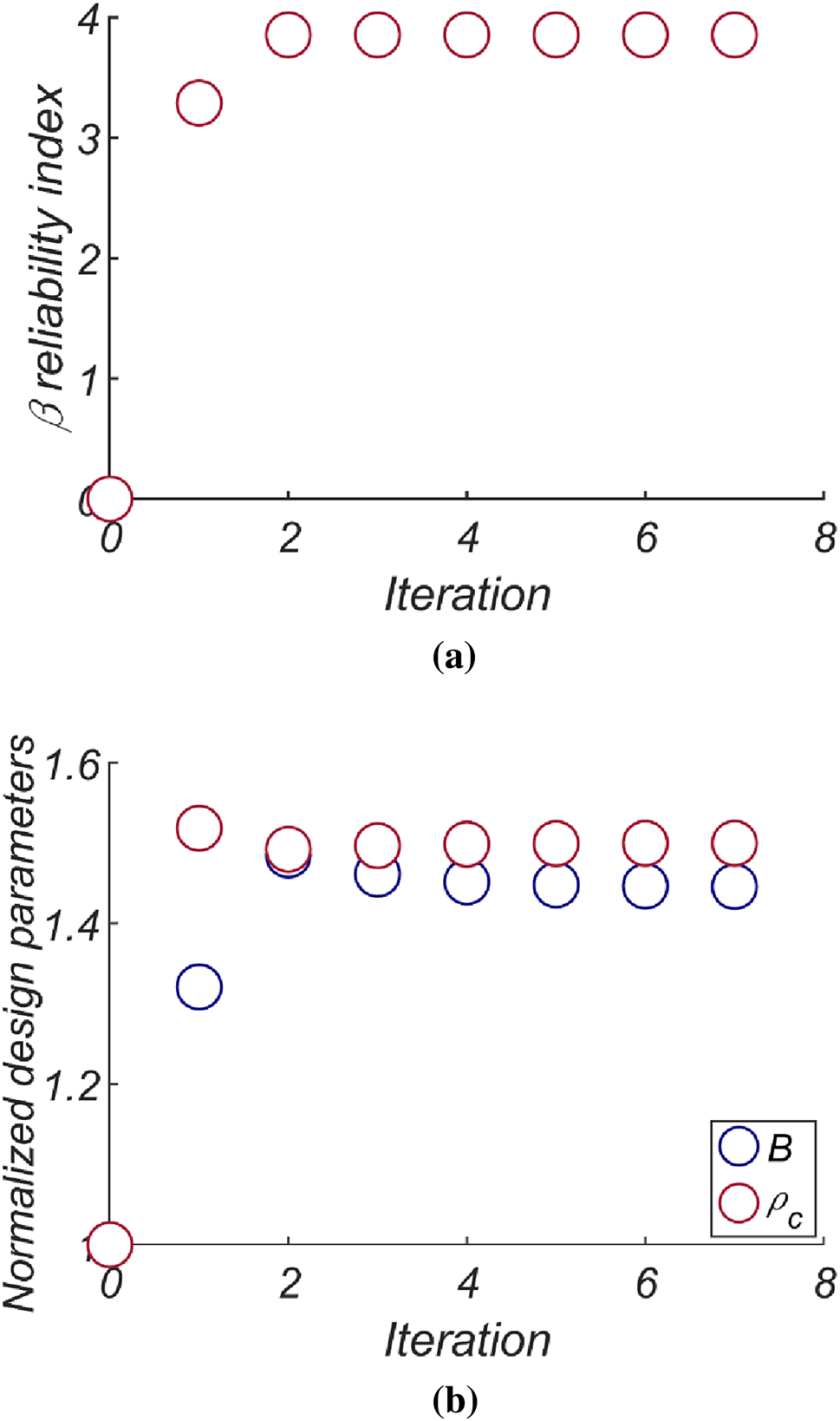
Variation of the reliability indices (**a**) $\beta$, (**b**) $B$, and $\rho_{C}$ as an iteration mechanism on.

The results of the reliability-based design optimization for the vertical-type breakwater, conducted by varying the target reliability index from 2 to 5, are presented in Table 6. Remarkably, as the target reliability index increases, the optimized vertical-type breakwater exhibits a significant increase in thickness. Conversely, a decrease in the target reliability index leads to a slight reduction in the weight of the optimized vertical-type breakwater. Notably, in RUN 5, the reliability constraint β = 5 proves to be unattainable, as the reliability index β = 4.6 approaches the limit state function described in Eq. (21) when B approaches its permissible limit of 40 m (refer to Eq. 25), thus serving as an upper bound. Figure 19 shows the evolution of the reliability index, breakwater thickness, and equivalent density during the optimization process, all of which are normalized by the initial guess. It is evident that as the errors introduced by the initial guess are progressively relaxed, the reliability index, breakwater thickness, and equivalent density quickly converge to their optimized values.

**Table 6:** Reliability-based design optimization results [HaeunDae].

	RUN 1	RUN 2	RUN 3	RUN 4	RUN 5
$\beta_{t}$	2	3	3.5	4	5
$N_{iter}$	7	7	7	7	7
$\beta$	2.00	3.0	3.5	4.0	4.6
$P[Z < 0]$	$2.26 \times 10^{ - 2}$	$1.3 \times 10^{ - 3}$	$2.321 \times 10^{ - 4}$	$3.167 \times 10^{ - 6}$	$1.743 \times 10^{ - 6}$
$B\;[{\text{m}}]$	12.23	17.35	21.84	28.65	42.48
$\rho_{C} \;[{\text{kg}}/{\text{m}}^{3} ]$	1964	2097	2100	2100	2066
$W_{optimized} \;[{\text{kg}}/{\text{m}}]$	$5.536 \times 10^{6}$	$8.374 \times 10^{6}$	$1.056 \times 10^{7}$	$1.385 \times 10^{7}$	$2.021 \times 10^{7}$
$N_{call}$	1684	3622	22,744	54,250	38,692

**Figure 20:**
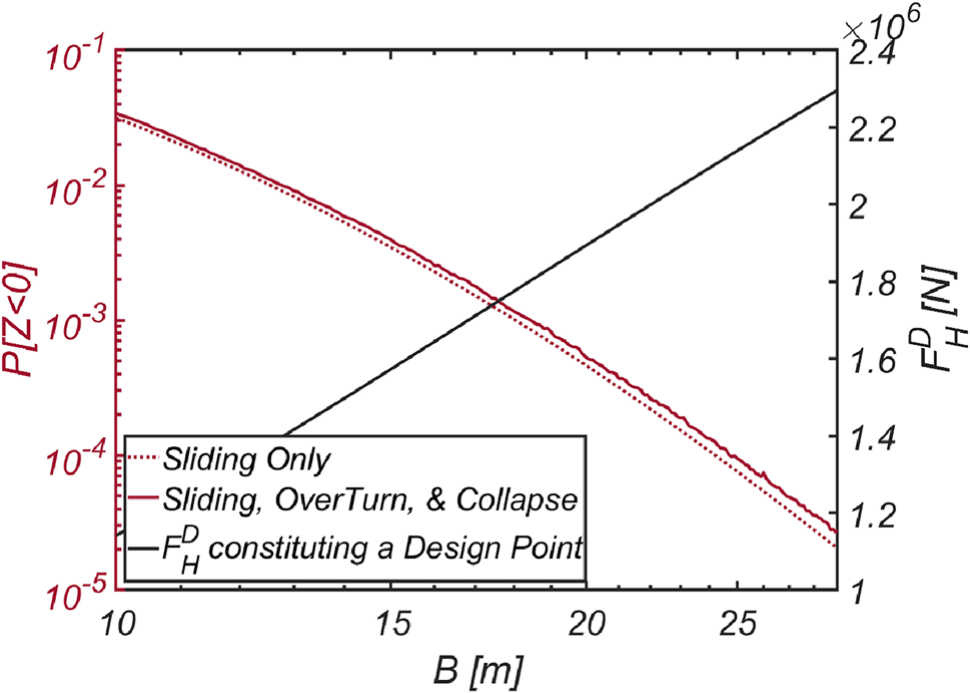
Variation in the failure probability of a vertical type breaker as the vertical type breakwater thickens with its associated $F_{H}^{D}$ constituting a design point for each $B$ [RUN 4, $\rho_{C} = 2100\;{\text{kg}}/{\text{m}}^{3}$].

In an effort to closely examine the validity of the reliability-based design optimization method presented in this study, the author conducted reliability analyses by varying the thickness of the vertical-type breakwater composed of filler ($\rho_{C} = 2100\;{\text{kg}}/{\text{m}}^{3}$, RUN4), which emerged during the optimization process, while considering three failure modes. Additionally, the author also carried out a reliability analysis using the FORM focused only on sliding failure to demonstrate how the overturning and collapse failure modes affect the safety of vertical-type breakwaters and gathered information about the wave force constituting the design point, $F_{H}^{D}$, along with the failure probability. The results of these reliability analyses are shown in Fig. 20. Within the context of the current reliability-based design platform in Korea, which primarily addresses sliding failure and focuses on the development of partial safety factors such as load and resistance coefficients based on design waves of a specific return period, the wave force at the design point, $F_{H}^{D}$, carries crucial information for evaluating partial safety factors and inferring a return period corresponding to the optimized vertical-type breakwater. This can serve as another metric for evaluating the performance of reliability-based design optimization method for vertical-type breakwaters presented in this study, other than failure probability. This perspective will be discussed in detail later in “On the robustness of design waves of specific return periods” section. In this context, Fig. 20 includes the wave force $F_{H}^{D}$ constituting the design point corresponding to each breakwater thickness as well. As expected, a thicker breakwater requires a greater wave force $F_{H}^{D}$ to initiate sliding. It is also evident that a thicker breakwater results in an increased lift force, significantly increasing the possibility of breakwater overturning failure.

#### Yeosu

The results of the reliability-based design optimization are listed in Table 7, showcasing the outcomes obtained by adjusting the target reliability indices $\beta_{t}$ to 2, 3, 3.5, 4, and 5. When comparing the optimized breakwater specifications with those in the sea off of Haeundae, the equivalent density of the vertical-type breakwater around [$\rho_{C} = 2100\;{\text{kg}}/{\text{m}}^{3}$] is found to be of a similar order. However, the vertical-type breakwater is considerably thicker in the sea off of Yeosu, indicating a more challenging marine environment. Notably, in RUN 10, the reliability constraint β = 5 is unattainable. Achieving this constraint would necessitate a bulky breakwater thicker than its permissible limit of 40 m [refer to Eq. 25], with the reliability index β = 4.08 serving as an upper bound.

**Table 7:** Reliability-based design optimization results [Yeosu].

	RUN 6	RUN 7	RUN 8	RUN 9	RUN 10
$\beta_{t}$	2	3	3.5	4	5
$N_{iter}$	7	7	7	7	7
$\beta$	2.0	3.0	3.51	3.95	4.08
$P[Z < 0]$	$2.27 \times 10^{ - 2}$	$1.3 \times 10^{ - 3}$	$2.14 \times 10^{ - 4}$	$3.7 \times 10^{ - 4}$	$2.2 \times 10^{ - 5}$
$B\;[{\text{m}}]$	13.77	22.78	29.65	42.65	46.03
$\rho_{C} \;[{\text{kg}}/{\text{m}}^{3} ]$	2076	2095	2100	2126	2132
$W_{optimized} \;[{\text{kg}}/{\text{m}}]$	$6.588 \times 10^{6}$	$1.099 \times 10^{7}$	$1.434 \times 10^{7}$	$2.088 \times 10^{7}$	$2.260 \times 10^{7}$
$N_{call}$	1594	3592	20,320	88,852	90,112

**Figure 21:**
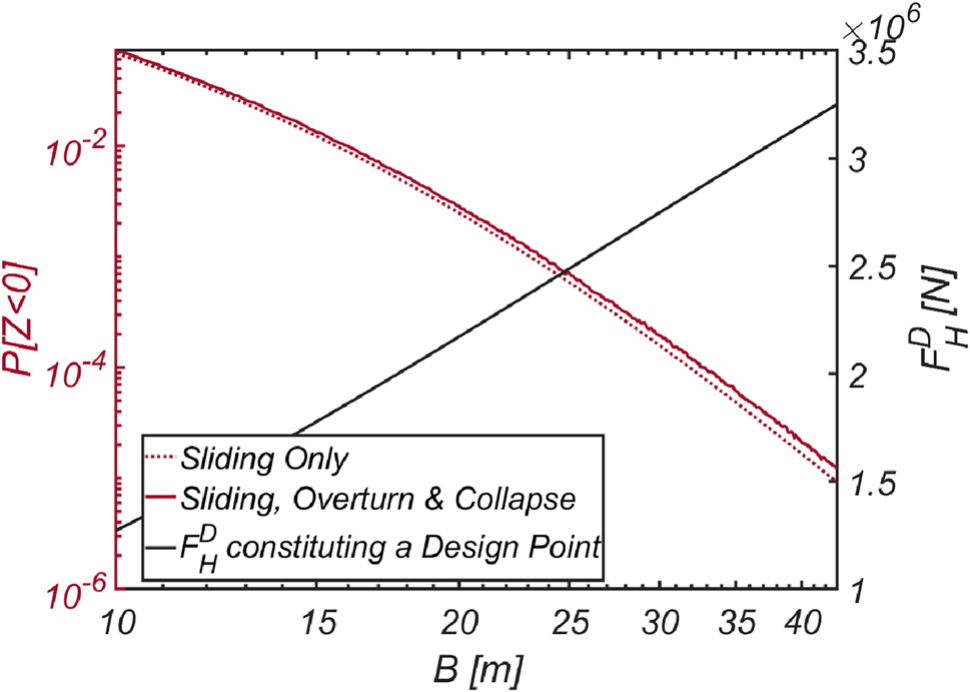
Variation in the failure probability of a vertical type breaker as the vertical type breakwater thickens with its associated $F_{H}^{D}$ constituting a design point for each $B$ [RUN 9, $\rho_{C} = 2126\;{\text{kg}}/{\text{m}}^{3}$].

To validate the reliability-based optimization design method presented in this study, similar to the approach taken for Haeundae, the author conducted reliability analyses considering three failure modes by adjusting the thickness of the vertical-type breakwater filled with optimized material ($\rho_{C} = 2126\;{\text{kg}}/{\text{m}}^{3}$, RUN 9). Additionally, reliability analyses using the FORM focused only on sliding failure were conducted for comparison with the current reliability-based design platform in Korea, which primarily addresses sliding failure and its associated partial safety factors. The results of these reliability analyses are shown in Fig. 21. As in the sea off of Haeundae, it is evident that the thicker the breakwater is, the greater the likelihood of breakwater overturning due to increased lift force.

#### Mokpo

**Figure 22:**
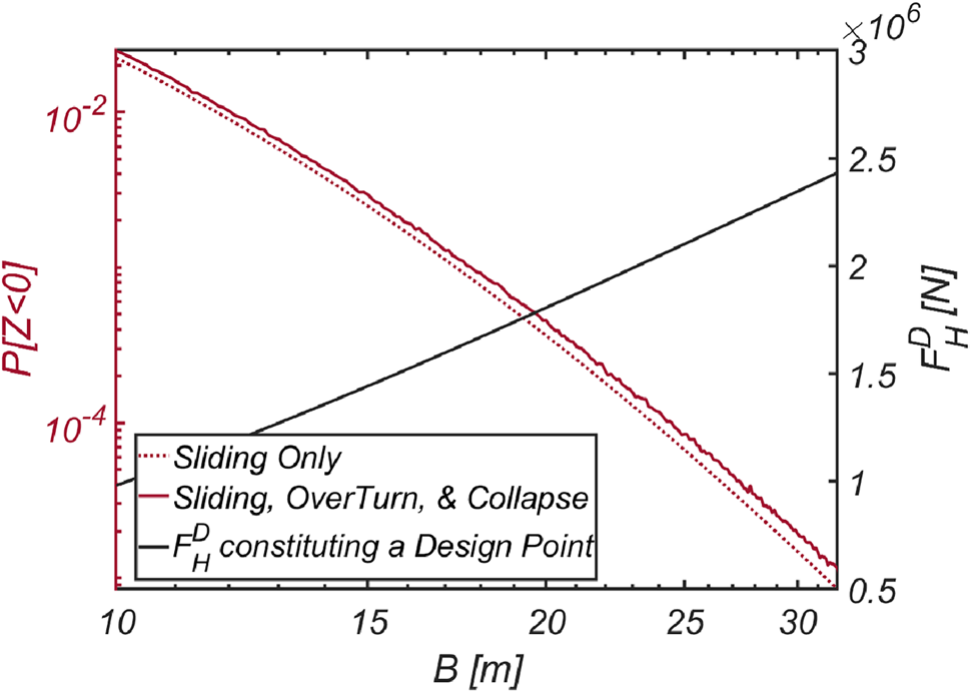
Variation in the failure probability of a vertical type breaker as the vertical type breakwater thickens with its associated $F_{H}^{D}$ constituting a design point for each $B$ [RUN 14, $\rho_{C} = 2099\;{\text{kg}}/{\text{m}}^{3}$].

The results of the reliability-based design optimization for the vertical-type breakwater, conducted by varying the target reliability index $\beta_{t}$ to 2, 3, 3.5, 4, and 5, are listed in Table 8. When comparing the optimized breakwater specifications in the sea off of MokPo to those in the sea off of Haeundae, the equivalent density $\rho_{C} = 2099\;{\text{kg}}/{\text{m}}^{3}$ and thickness $B$ of the vertical-type breakwater are of a similar order. This suggests that the marine environment in the sea off of MokPo is similar to that in the sea off of Haeundae in terms of roughness. Similar to Haeundae and Yeosu, the reliability constraint β = 5 [RUN 15] is unattainable, with the reliability index β = 4.54 serving as an upper bound. The reliability analysis results, considering various combinations of failure modes and obtained by varying the thickness of the vertical-type breakwater filled with material ($\rho_{C} = 2099\;{\text{kg}}/{\text{m}}^{3}$, RUN14) identified using the optimization process, are presented in Fig. 22. As in Haeundae and Yeosu, it is evident that the thicker the caisson is, the greater the lift force, which increases the possibility of breakwater overturning.

**Table 8:** Reliability-based optimization results [Mokpo].

	RUN 11	RUN 12	RUN 13	RUN 14	RUN 15
$\beta_{t}$	2	3	3.5	4	5
$N_{iter}$	7	7	7	7	7
$\beta$	2.0	3.00	3.5	4.21	4.54
$P[Z < 0]$	$2.274 \times 10^{ - 2}$	$1.34 \times 10^{ - 3}$	$2.292 \times 10^{ - 4}$	$1.256 \times 10^{ - 5}$	$2.812 \times 10^{ - 6}$
$B\;[{\text{m}}]$	10.26	16.60	21.33	31.51	41.77
$\rho_{C} \;[{\text{kg}}/{\text{m}}^{3} ]$	2066	2094	2097	2099	2109
$W_{optimized} \;[{\text{kg}}/{\text{m}}]$	$4.885 \times 10^{6}$	$8.011 \times 10^{6}$	$1.030 \times 10^{7}$	$1.524 \times 10^{7}$	$2.029 \times 10^{7}$
$N_{call}$	2506	3886	8314	37,486	88,876

#### Gunsan

**Figure 23:**
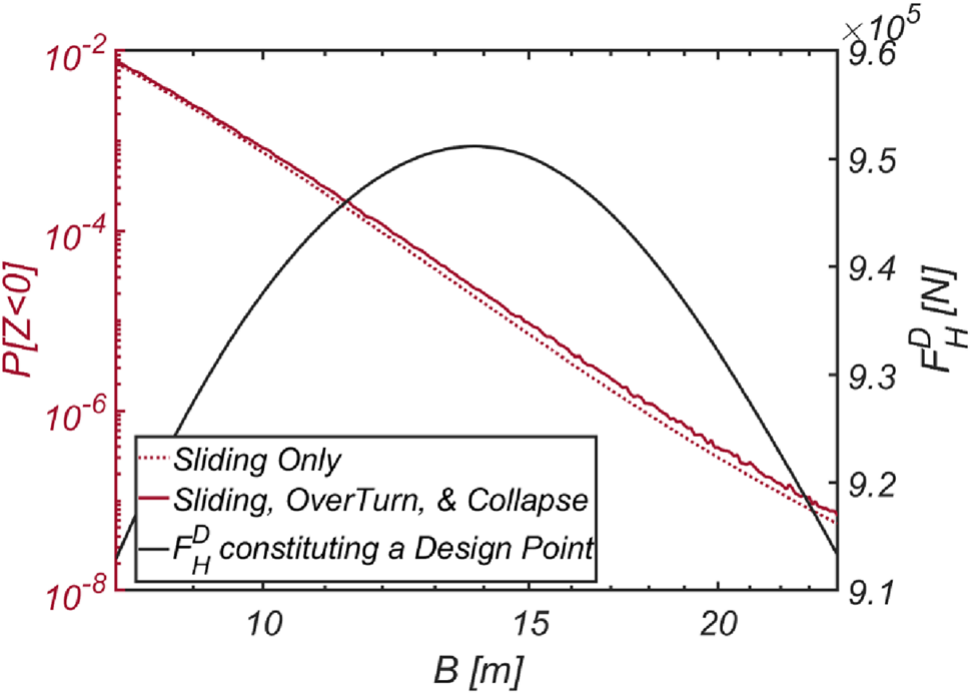
Variation in the failure probability of a vertical type breaker as the vertical type breakwater thickens with its associated $F_{H}^{D}$ constituting a design point for each $B$ [RUN 19, $\rho_{C} = 2100\;{\text{kg}}/{\text{m}}^{3}$].

The results of the reliability-based optimization design, conducted while varying the target reliability index $\beta_{t}$ to 2, 3, 4, and 5, are listed in Table 9. When comparing the optimized breakwater specifications with those in the sea off of Haeundae and Mokpo, the equivalent density of the vertical-type breakwater at approximately $\rho_{C} = 2100\;{\text{kg}}/{\text{m}}^{3}$ is shown to be of a similar order. However, the thickness of the vertical-type breakwater is only half that of the previously studied sea area, suggesting that the marine environment in the sea off of Gunsan is relatively mild. The reliability analysis results, considering various combinations of failure modes and obtained by varying the thickness of the vertical-type breakwater filled with material ($\rho_{C} = 2100\;{\text{kg}}/{\text{m}}^{3}$, RUN 19), identified using the optimization process, are presented in Fig. 23 to closely examine the validity of the reliability-based optimization design method presented in this study. As in Haeundae, Yeosu, and Mokpo, it is evident that the thicker the caisson is, the greater the lift force, which increases the possibility of overturning a breakwater.

**Table 9:** Reliability-based optimization results [Gunsan].

	RUN 16	RUN 17	RUN 18	RUN 19	RUN 20
$\beta_{t}$	2	3	3.5	4	5
$N_{iter}$	7	7	7	7	7
$\beta$	2.0028	3.001	3.645	4.007	5.0
$P[Z < 0]$	$2.25 \times 10^{ - 2}$	$1.340 \times 10^{ - 3}$	$1.334 \times 10^{ - 4}$	$3.071 \times 10^{ - 5}$	$2.323 \times 10^{ - 7}$
$B\;[{\text{m}}]$	10.00	10.00	12.18	13.75	21.90
$\rho_{C} \;[{\text{kg}}/{\text{m}}^{3} ]$	1910	2077	2096	2100	2059
$W_{optimized} \;[{\text{kg}}/{\text{m}}]$	$4.399 \times 10^{6}$	$4.786 \times 10^{6}$	$5.833 \times 10^{6}$	$6.652 \times 10^{6}$	$1.039 \times 10^{7}$
$N_{call}$	6076	30,106	40,762	38,374	31,672

One interesting phenomenon here is that after reaching its peak at $B = 13.5\;{\text{m}}$, the wave force $F_{H}^{D}$ at which the vertical-type breakwater begins to slide decreases as the breakwater becomes thicker. This implies that thicker breakwaters can initiate sliding even with relatively small wave forces, which warrants further discussion. This seemingly awkward stochastic behavior has a solid physical foundation when considering that the failure probability decreases as the breakwater becomes thicker. The occurrence of damage to a massive breakwater through sliding, overturning, or collapse demands abnormally high tides and a low friction coefficient, even though the likelihood of such conditions is very low. With these exceptionally large buoyant forces and small friction forces, the resistance of the breakwater to sliding and overturning is reduced, increasing susceptibility to sliding or overturning even with small wave forces. Given that this stochastic behavior aligns precisely with our physical intuition, it can be concluded that the reliability analysis and reliability-based design optimization conducted in this study were performed with a high degree of accuracy.

#### Incheon

**Figure 24:**
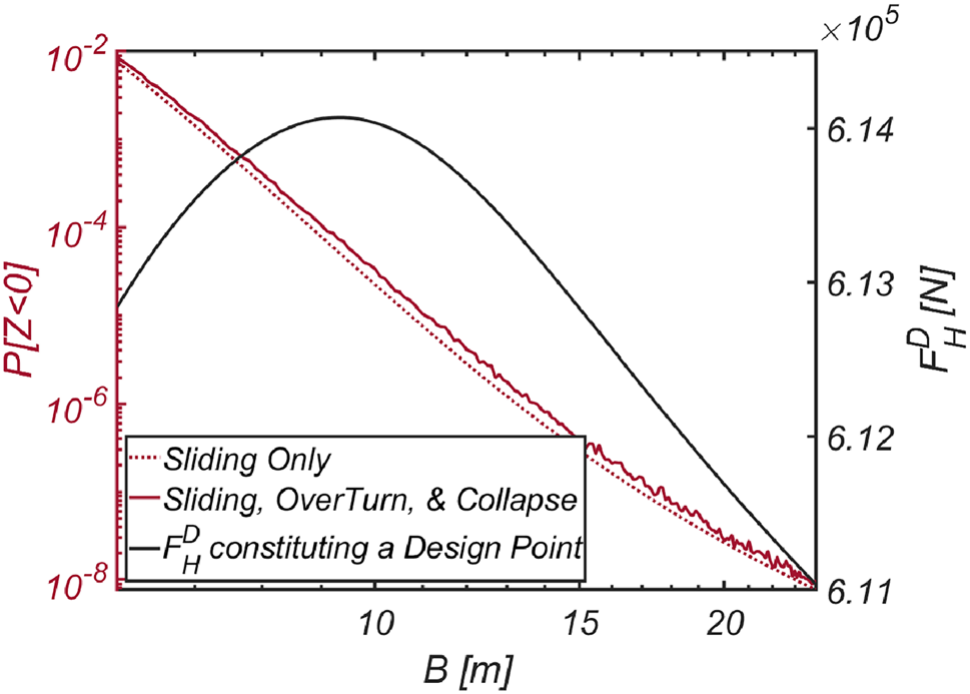
Variation in the failure probability of a vertical type breaker as the vertical type breakwater thickens with its associated $F_{H}^{D}$ constituting a design point for each $B$ [RUN 24, $\rho_{C} = 2046\;{\text{kg}}/{\text{m}}^{3}$].

The results of the reliability-based optimization design for the vertical-type breakwater, conducted by varying the target reliability index $\beta_{t}$ to 2, 3, 4, and 5, are listed in Table 10. The equivalent density and thickness of the optimized breakwater were simulated to be $\rho_{C} = 2046\;{\text{kg}}/{\text{m}}^{3}$ and $B = 10.24\;{\text{m}}$, respectively, which are the lowest among the sea areas discussed in this study. This indicates that the marine environment in the sea off of Incheon is the mildest. The reliability analysis results, considering various combinations of failure modes and obtained by varying the thickness of the vertical-type breakwater filled with optimized material ($\rho_{C} = 2046\;{\text{kg}}/{\text{m}}^{3}$, RUN24), are presented in Fig. 24 to closely examine the validity of the reliability-based optimization design presented in this study. Like in Haeundae, Yeosu, Mokpo, and Gunsan, it is evident that thicker caissons result in greater lift forces, increasing the likelihood of breakwater overturning. However, in the sea off of Incheon, there is an intriguing observation: the critical wave force at which the vertical-type breakwater begins to slide decreases as the thickness of the breakwater surpasses a certain threshold. This somewhat awkward stochastic phenomenon is believed to be influenced by abnormally high tide levels and a low friction coefficient, both of which have a very low likelihood of occurrence, as observed in the sea off of Gunsan.

**Table 10:** Reliability-based optimization results [Incheon].

	RUN 21	RUN 22	RUN 23	RUN 24	RUN 25
$\beta_{t}$	2	3	3.5	4	5
$N_{iter}$	7	7	7	7	7
$\beta$	2.407	3.015	3.549	4.014	5.000
$P[Z < 0]$	$8 \times 10^{ - 3}$	$1.28 \times 10^{ - 3}$	$1.926 \times 10^{ - 4}$	$2.973 \times 10^{ - 5}$	$2.796 \times 10^{ - 7}$
$B\;[{\text{m}}]$	10.02	10.03	10.02	10.24	14.30
$\rho_{C} \;[{\text{kg}}/{\text{m}}^{3} ]$	1725	1833	1942.58	2046.29	2099
$W_{optimized} \;[{\text{kg}}/{\text{m}}]$	$3.984 \times 10^{6}$	$4.235 \times 10^{6}$	$4.387 \times 10^{6}$	$4.829 \times 10^{6}$	$6.919 \times 10^{6}$
$N_{call}$	38,440	37,216	44,356	35,998	14,260

## On the robustness of design waves of specific return periods

If the return period of the design waves used in the deterministic design of a vertical-type breakwater is appropriate, the failure probability and physical specifications of the breakwater designed based on that return period should align with the results achieved through optimization. From this perspective, the appropriate return period can be estimated from the yearly maximum wave force constituting a design point corresponding to the thickness of the optimized vertical-type breakwater and the yearly maximum wave force probability distribution. This approach provides an opportunity to address long-standing and controversial issues within the Korean coastal engineering community. One such question is whether design waves with a 50- or 100-year return period can accurately depict the inherent irregularities in random variables such as wave force, lift force, tidal level, friction coefficient, and overturning moment acting on vertical-type breakwaters. Consequently, this inverse estimation of the appropriate return period holds significant engineering value.

**Figure 25:**
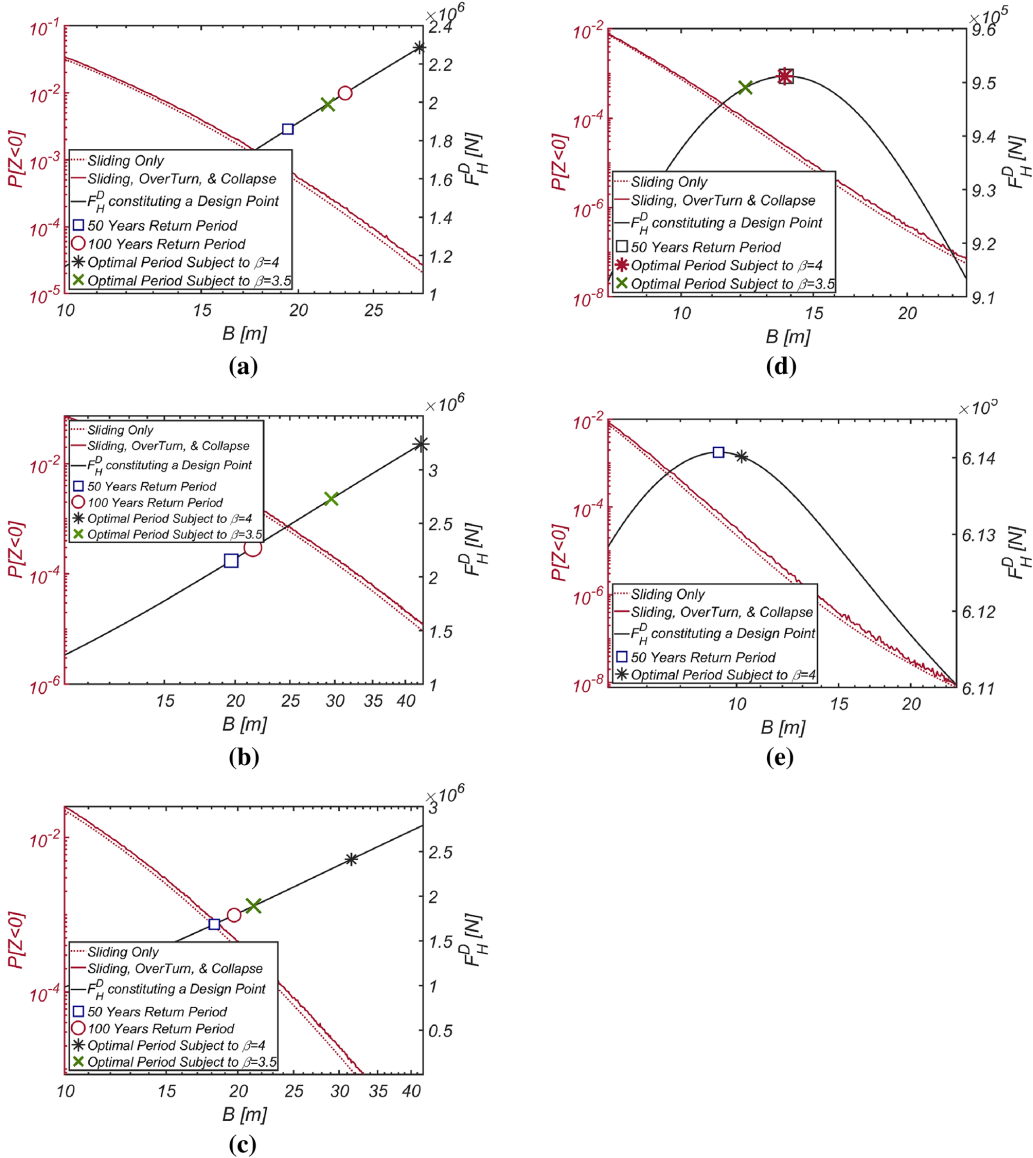
Variation in the failure probability of a vertical type breaker as the vertical type breakwater thickens with associated $F_{H}^{D}$ constituting a design point for each $B$. (**a**) Haeundae [RUN 3, $\rho_{C} = 2100\;{\text{kg}}/{\text{m}}^{3}$ and β = 3.5], (**b**) Yeosu [RUN 8, $\rho_{C} = 2100\;{\text{kg}}/{\text{m}}^{3}$ and β = 3.5], (**c**) Mokpo [RUN 13, $\rho_{C} = 2097\;{\text{kg}}/{\text{m}}^{3}$ and β = 3.5], (**d**) Gunsan [RUN 18, $\rho_{C} = 2096\;{\text{kg}}/{\text{m}}^{3}$ and β = 3.5], (**e**) Incheon [RUN 24, $\rho_{C} = 2046\;{\text{kg}}/{\text{m}}^{3}$ and β = 4.0].

To assess the robustness of the design return period, Fig. 25 illustrates how the failure probability varies as the vertical-type breakwater for β = 3.5 becomes thicker. Among the random variables that constitute the design point for each thickness, Fig. 25 includes the wave forces $F_{H}^{D}$, $F_{H}^{50\;Years}$, and $F_{H}^{100\;Years}$, each with return periods of 50 years and 100 years. Here, $F_{H}^{50\;Years}$ and $F_{H}^{100\;Years}$ defined by 2% and 1% exceedance probabilities, respectively, were estimated using the yearly maximum wave force probability distribution presented in this study. For clarity, the author also included the wave force $F_{H}^{D}$ corresponding to the breakwater thickness optimized to achieve a reliability index of β = 3.5 and 4.

First and foremost, it is evident that the failure probability of a breakwater designed based on a specific return period drastically varies across all sea areas considered in this study. In the seas off of Haeundae, Yeosu, and Mokpo, which are exposed to harsh marine environments, even when adopting a wave force $F_{H}^{100\;Years}$ with a 100-year return period as the design wave force, it is not feasible to attain the target failure probability corresponding to the reliability index β = 3.5–4. This result implies that conventional deterministic design practices have led to the construction of a breakwater that is significantly under-designed. These simulation results explain the recent damage experienced by many vertical-type breakwaters deployed along the southern coast of Korea. These breakwaters were originally designed to withstand the very harsh waves resulting from climate change; these harsh waves were modeled as waves with a 100-year return period. On the other hand, in the seas off of Gunsan and Incheon, where the marine environment is relatively mild, simulation results show that it is possible to design a highly resilient vertical-type breakwater even with wave forces $F_{H}^{50\;Years}$ of a 50-year return period. In contrast, a reliability-based optimization design targeting a reliability index of β = 3.5–4 ensures a consistent failure probability across all sea areas.

**Figure 26:**
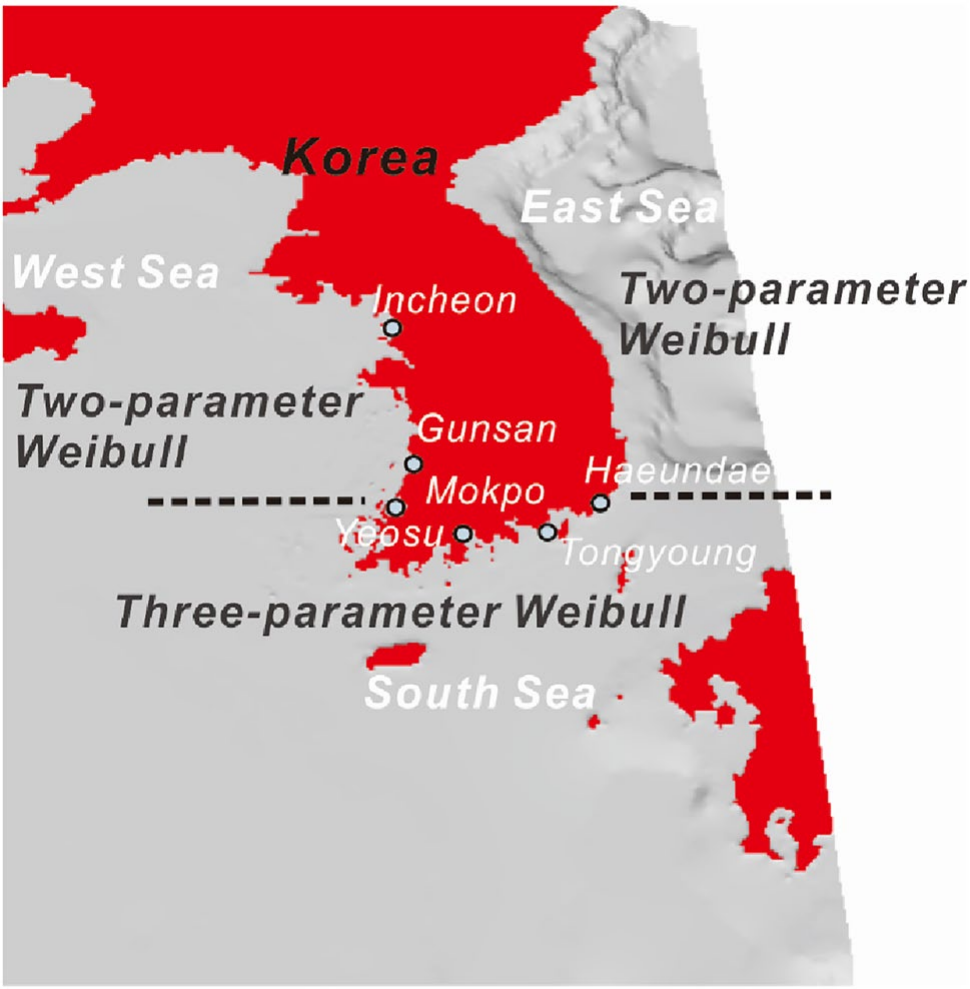
Variation in the optimized probabilistic model along the coast of the Korean Peninsula. The map was created using MIKE 21, a software developed by DHI Group, available at https://www.mikepoweredbydhi.com/products/mike-21-3.

Many factors contribute to the discrepancies between the design wave approach and reliability-based design optimization approach, with deficiencies in concepts such as design waves of a 50- or 100-year return period standing out the most. As discussed earlier, design waves of specific return periods simply serve as a means of defining the environmental load acting on the breakwater for the convenience of designers and do not offer insight into the robustness of a breakwater designed to withstand such waves. This perspective is confirmed by the inconsistent failure probability estimated using design waves of specific return periods, which are evaluated based on the yearly maximum probability distribution derived from the local in-situ wave data. Therefore, if design waves of specific return periods are a legitimate tool, they should yield consistent failure probabilities across all sea areas considered in this study because local wave conditions are already fully incorporated in the yearly maximum probability distribution presented. Additionally, overlooked overturning and collapse might also contribute. However, as discussed earlier, the contribution of overlooked overturning and collapse is around 5%, and these overlooked failure modes alone cannot fully explain the discrepancies between the design wave approach and reliability-based design optimization approach. Considering the varying simulation results across different sea areas, it can be concluded that solely relying on design waves with specific return periods to describe Korea's diverse marine environments, each with unique characteristics (refer to Fig. 26), would lead to a poorly designed breakwater. Therefore, there is a clear need to introduce an optimal design approach based on reliability that satisfies the target reliability index β = 3.5–4. The load coefficients for design waves with return periods of 50 and 100 years, with β=4.0, are listed in Tables 11, 12, 13, 14 and 15.

**Table 11:** List of load coefficients for design waves of return periods of 50 and 100 years at Haeundae for β = 4.0.

	Load coefficients	$B\;[{\text{m}}]$	$P[Z < 0]$
50 Year return period	1.229	19.4	$6.69 \times 10^{ - 4}$
100 Year return period	1.116	23.0	$1.89 \times 10^{ - 4}$

**Table 12:** List of load coefficients for design waves of return periods of 50 and 100 years at Yeosu for β = 4.0.

	Load coefficients	$B\;[{\text{m}}]$	$P[Z < 0]$
50 Year return period	1.505	19.7	$3.114 \times 10^{ - 3}$
100 Year return period	1.424	21.5	$1.812 \times 10^{ - 3}$

**Table 13:** List of load coefficients for design waves of return periods of 50 and 100 years at Mokpo for β = 4.0.

	Load coefficients	$B\;[{\text{m}}]$	$P[Z < 0]$
50 Year return period	1.431	18.2	$8.381 \times 10^{ - 4}$
100 Year return period	1.349	19.7	$4.99 \times 10^{ - 4}$

**Table 14:** List of load coefficients for design waves of return periods of 50 and 100 years at Gunsan for β = 4.0.

	Load coefficients	$B\;[{\text{m}}]$	$P[Z < 0]$
50 Year return period	1.0	13.7	$2.51 \times 10^{ - 5}$

**Table 15:** List of load coefficients for design waves of return periods of 50 and 100 years at Incheon for β = 4.0.

	Load coefficients	$B\;[{\text{m}}]$	$P[Z < 0]$
50 Year return period	0.99	9.3	$7.389 \times 10^{ - 5}$

## Conclusion

Within the Korean coastal engineering community, reliability-based design has long been considered an alternative for overcoming the limitations of deterministic design. Despite extensive efforts over the past decade, its application in design practice has remained somewhat limited.

Several factors contribute to the limited application of reliability-based design in Korea. The current reliability-based design platform in Korea predominantly focuses on the failure probability of vertical-type breakwaters related to sliding, overlooking crucial aspects such as overturning and collapse due to exceeding the allowable bearing capacity of their rock foundation. This limitation hampers the broader adoption of reliability-based design. Additionally, the omission of physical properties, such as the total amount of filler used in vertical-type breakwaters and its specific weight, further impedes the integration of reliability-based design into practice. A persistent and controversial issue within the Korean coastal engineering community revolves around whether design waves with return periods of 50 or 100 years adequately account for inherent irregularities in random variables, such as wave and lift forces, as well as overturning moments acting on the breakwaters. These concerns, coupled with the lack of probabilistic models for capturing the varied characteristics of the Korean marine environment from sea to sea, further hinder the application of reliability-based design. It is worth noting that these concerns have consistently been raised within certain factions of the Korean coastal engineering community.

In this study, with the aim of promoting the application of reliability-based design within the Korean coastal engineering community, the author conducted reliability analyses and reliability-based design optimization of vertical-type breakwaters considering multiple limit states. This study focused on the ports in Haeundae, Yeosu, Mokpo, Gunsan, and Incheon, which are representative ports in Korea. In this process, to perform a comprehensive reliability analysis, the author deliberately abstained from using design waves of a specific return period. Instead, the author characterized the uncertainties associated with the wave force, lift force, and overturning moment—these probabilistic variables influence the integrity of the vertical-type breakwater—by employing a probabilistic model derived directly from long-term in situ wave data collected hourly. The limit state of the vertical type breakwater was composed of three failure modes, sliding, overturning, and collapse, and the close relationships between the wave force, lift force, and moment were described using the Nataf joint probability distribution. Furthermore, to assess the robustness of the design waves underpinning Korea's reliability-based design platform for vertical type breakwaters using the partial safety factor method, reverse engineering is used to determine appropriate return periods based on the physical properties of the optimally designed vertical breakwater and the probability distribution of the yearly maximum wave force.

As anticipated, the simulation results showed that the failure probability is underestimated when considering only sliding failure. When accounting for collapse due to foundation damage, the failure probability of the vertical breakwater increased to some extent. This trend became more pronounced as the thickness of the vertical breakwater increased. In the case of the optimized vertical-type breakwater, the failure probability due to overturning evidently increased with increasing thickness, primarily because of the increased lifting force. The contribution from the collapse of the breakwater, the weight of which was reduced through the optimization process, was negligible. These simulation results align with our physical intuition. If sliding and overturning are treated as mutually independent failure modes, as in past studies, it becomes evident that vertical-type breakwaters are currently underdesigned, highlighting the need for urgent amendment. Furthermore, the failure probability of vertical-type breakwaters cannot be consistently ensured using design waves with a specific return period. In contrast, breakwaters optimally designed to meet the reliability index requirement of β = 3.5–4 have been shown to achieve a consistent probability of destruction across all sea areas. The varying simulation results across different sea areas strongly indicate the challenge of relying on design waves with specific return periods to describe Korea’s diverse marine environments, each with unique characteristics. Therefore, there is a clear need to introduce an optimal design approach based on reliability that satisfies the target reliability index β = 3.5–4. Furthermore, the probabilistic models developed in this study do not require any additional assumptions regarding the relationship between significant wave and maximum wave heights, along with the wave period, as in the study by Castillo et al. (2006). Following Occam's razor principle, which suggests that explanations constructed with the smallest possible set of assumptions are superior, the reliability-based design optimization of a vertical-type breakwater presented in this study demonstrates promise in terms of simplicity and practicality.

## Data Availability

The datasets used and/or analysed during the current study available from the corresponding author on reasonable request.
